# STEAP3 Inhibits Porcine Reproductive and Respiratory Syndrome Virus Replication by Regulating Fatty Acid and Lipid Droplet Synthesis

**DOI:** 10.3390/vetsci12020147

**Published:** 2025-02-08

**Authors:** Chenyang Yuan, Kaifeng Guan, Gaiping Zhang

**Affiliations:** 1 College of Veterinary Medicine, Northwest A&F University, Yangling 712100, China; yuanchenyang@nwafu.edu.cn; 2 International Joint Research Center of National Animal Immunology, College of Veterinary Medicine, Henan Agricultural University, Zhengzhou 450046, China; 3 School of Advanced Agricultural Sciences, Peking University, Beijing 100871, China

**Keywords:** STEAP3, PRRSV, transcriptomics, metabolomics, lipogenesis

## Abstract

Porcine Reproductive and Respiratory Syndrome (PRRS) is a contagious disease that impacts swine health worldwide. Lipid metabolism plays a vital role in energy production and is regulated by various genes involved in lipogenesis and lipolysis. In this study, we found that PRRSV infection significantly reduced the protein expression of STEAP3. The overexpression of STEAP3 can notably inhibit PRRSV replication. Additionally, we utilized transcriptomics and metabolomics to examine the effects of STEAP3 on PRRSV replication, identifying important pathways associated with energy metabolism and lipogenesis. We subsequently found that STEAP3 can suppress PRRSV replication by regulating fatty acid synthesis and enhancing lipid droplet formation. Overall, these findings indicate that STEAP3 could be a potential target for developing strategies to manage PRRSV infection by modulating lipid metabolism.

## Simple Summary

Porcine Reproductive and Respiratory Syndrome (PRRS) caused by PRRSV has caused significant economic losses to the pig industry. Focusing on host antiviral genes and exploring the interaction between PRRSV and the host are crucial to understand the pathogenesis of PRRSV. This study takes STEAP3, which is significantly downregulated after PRRSV infection, as the entry point and finds that lipid metabolism plays a key role in the replication of PRRSV. We found that STEAP3 inhibits PRRSV replication by regulating fatty acid and lipid droplet synthesis. This provides a new strategy for PRRS prevention and control.

## 1. Introduction

Porcine Reproductive and Respiratory Syndrome (PRRS) represents a highly contagious animal disease that significantly affects swine health on a global scale [1]. The causative agent, Porcine Reproductive and Respiratory Syndrome Virus (PRRSV), is a single-stranded positive-sense RNA virus characterized by a genome approximately 15 KB in length that encodes various structural and non-structural proteins [2]. Infection with PRRSV can result in respiratory complications in piglets and reproductive challenges in sows, leading to substantial economic repercussions for the swine industry annually [1]. Given its inability to independently metabolize, the virus is dependent on host cellular mechanisms to complete its lifecycle [3]. Following viral infection, the metabolic processes of host cells can be modulated to either facilitate viral persistence or promote its elimination. Research has indicated that PRRSV infection can induce modifications in the lipid metabolism of host cells, and conversely, changes in host lipid metabolism can influence the replication of PRRSV [4]. Consequently, investigating the interplay between host lipid metabolism and PRRSV replication is crucial for comprehending virus replication strategies and for developing effective prevention and control measures.

Lipid metabolism constitutes a critical cellular metabolic process that is modulated by a variety of essential genes associated with lipid metabolism. These include genes involved in lipogenesis, such as Acetyl-CoA Carboxylase 1 (ACC1), Fatty Acid Synthase (FASN), and Stearoyl-CoA Desaturase-1 (SCD-1), as well as those implicated in lipolysis, including Adipose Triglyceride Lipase (ATGL), Hormone-Sensitive Lipase (HSL), and Monoglyceride Lipase (MGL) [5,6]. As a primary source of energy within cells, lipid metabolism significantly influences the viral replication cycle [7,8]. Empirical studies have demonstrated that Dengue Virus (DENV) infection leads to the release of free fatty acids (FFA) through the degradation of lipid droplets (LDs), thereby generating ATP that facilitates viral replication [9]. Furthermore, Sterol Regulatory Element Binding Protein (SREBP), a pivotal regulatory factor for lipogenesis, is upregulated following Hepatitis C Virus (HCV) infection, which in turn enhances viral replication [10]. Additionally, the PRRSV is also dependent on the host’s lipid metabolic pathways for its replication [11]. Research findings suggest that the deletion of Sphingomyelin Phosphodiesterase Acid-Like 3B (SMPDL3B) can effectively impede PRRSV infection by modulating lipid metabolism [12]. Moreover, Yin Yang 1 (YY1) has been shown to inhibit PRRSV replication through the regulation of lipid metabolism and the reprogramming of intracellular lipid droplet synthesis [3].

The STEAP (Six-Transmembrane Epithelial Antigen of the Prostate) family comprises a distinct class of transmembrane proteins that function as reductive enzymes exclusive to mammals. These proteins are integral to redox reactions within the organism and are implicated in various metabolic disorders, including obesity, insulin resistance, and cardiac hypertrophy [13,14]. Among this family, STEAP3 serves as the principal intracellular reductase and is involved in numerous biological processes. STEAP3 facilitates the reduction in intracellular ferric ions to ferrous ions, subsequently promoting lipid peroxidation and cellular ferroptosis via the Fenton reaction [15,16]. The absence of STEAP3 has been shown to impede the progression of non-alcoholic fatty liver disease (NAFLD), whereas its overexpression can lead to lipid accumulation within hepatic cells [17]. Furthermore, STEAP3 has been demonstrated to inhibit the replication of human cytomegalovirus [18]. Considering the significant association between STEAP3 and lipid metabolism, as well as the critical role of lipid metabolism in viral replication, we posited that STEAP3 might regulate the replication of PRRSV through the modulation of lipid metabolism. In our investigation, we observed a marked decrease in the protein expression levels of STEAP3 over time following PRRSV infection. Notably, the overexpression of STEAP3 had a substantial impact on lipid metabolism, ultimately leading to the inhibition of PRRSV replication, which was mediated by the regulation of fatty acid and LD synthesis.

## 2. Materials and Methods

**Cell, Virus, Plasmids, and Transfections**

MARC-145 cells (monkey embryonic kidney epithelial cells) were obtained from the Key Laboratory of Animal Immunology at the Henan Academy of Agricultural Sciences and maintained in DMEM (Dulbecco’s modified Eagle medium) (Solarbio, Beijing, China), supplemented with 10% FBS (fetal bovine serum) (TransGen Biotech, Beijing, China). The HP-PRRSV heN-07 strain, and the pcDNA3.1 plasmid, are all derived from the above laboratory. The pcDNA3.1-STEAP3 and the empty pcDNA3.1 vector were individually introduced into MARC-145 cells via electroporation utilizing GenePulser Xcell (Bio-Rad, Richmond, CA, USA). The electroporation was conducted at a voltage of 180V with a capacitance of 950 μF. The cells were transferred to culture plates and then infected with PRRSV (MOI = 1) after being cultured for 12 h. The cells were collected at different times post-infection.

**Western blot (Original Images in the Supplementary Materials)**

Cell samples were lysed using RIPA (Beyotime, Shanghai, China) to extract total protein. Following denaturation, the proteins were subjected to separation via SDS-PAGE and subsequently transferred to PVDF membrane (Millipore, MA, USA). The membrane was then blocked with a 5% non-fat milk solution for a duration of 1–2 h. The primary antibody (Anti-STEAP3 antibody, Abcam, Cambridge, UK, ab151566, 1:1000; PRRS virus Nucleocapsid protein antibody, GeneTex, Irvine, CA, USA, GTX637650, 1:1000; GAPDH Polyclonal antibody, Proteintech, 10494-1-AP, 1:10,000), prepared in PBST, was incubated with the membrane overnight at 4 °C. After the removal of the primary antibody, the membrane was washed three times for 10 min each. The secondary antibody (HRP-conjugated Goat Anti-Rabbit IgG(H+L), Proteintech, Wuhan, China, SA00001-2, 1:10,000), also diluted in PBST, was applied and incubated at room temperature for 1 h. Following three additional washes, the membrane was developed using ECL chemiluminescent reagent (Beyotime, Shanghai, China).

**Quantitative Real-Time PCR**

Total RNA from the cells was extracted using RNAiso Plus (TAKARA, Tokyo, Japan), followed by reverse transcription to obtain cDNA samples. Quantitative PCR was performed using cDNA as the template to assess the mRNA levels of the relevant genes. The reaction mixture consisted of the following components: 5 µL of SYBR Green, 3.2 µL of dd H_2_O, 0.4 µL of upstream primer, 0.4 µL of downstream primer, and 1 µL of cDNA. The specific quantitative primers used are detailed in Table 1.

**Transmission Electron Microscope (TEM)**

MARC-145 cells were infected with PRRSV that overexpresses STEAP3, alongside the establishment of a negative control group. Following a 24-h incubation period, the cells were fixed using glutaraldehyde (Solarbio, Beijing, China). Transmission electron microscopy (HITACHI H-7000FA, Tokyo, Japan) was then conducted to assess the morphology, size, and quantity of the mitochondria, which were subsequently analyzed statistically.

**RNA-seq and Analysis**

MARC-145 cells with or without STEAP3 overexpression were infected with PRRSV, and total RNA was extracted from the cells 24 h post-infection. The data were quality-controlled using fastp (version 0.23.0) software to obtain high-quality clean data. Subsequently, the clean data were aligned to the *Chlorocebus sabaeus* reference genome (ChlSab1.1_release108) to obtain comprehensive transcript information. Finally, transcriptome analysis was performed, including differential expression gene screening and functional pathway analysis of differentially expressed genes.

**Metabolomics and Analysis**

MARC-145 cells with or without STEAP3 overexpression were infected with PRRSV, and cell samples were collected 24 h post-infection. All small-molecule metabolites in the samples were detected using a high-resolution LC-MS/MS platform. After constructing the metabolome library and quality control, the identified metabolites were annotated and analyzed using the HMDB and KEGG databases. Fisher’s exact test and GSEA method were employed to calculate significantly enriched pathways of differentially abundant metabolites.

**Flow Cytometry Analysis**

MARC-145 cells were seeded in a 6-well plate, and then STEAP3 overexpression was induced in these cells before infecting them with PRRSV. After 24 h, the cells were trypsinized and resuspended in PBS. Then, the cells were treated with the BODIPY 493/503 Staining Kit (Beyotime, Shanghai, China) and the fluorescence intensity of the lipid droplets was measured using a flow cytometer.

**Microscopy**

PRRSV-infected MARC-145 cells with or without STEAP3 overexpression were stained using the BODIPY 493/503 Staining Kit (Beyotime, Shanghai, China). Subsequently, green puncta of LDs were observed on confocal microscopy (Zeiss LSM 800, Oberkochen, Germany).

**Statistical Analysis**

Data analysis and plotting were performed using GraphPad Prism 10.1.2 software, and significance difference analysis was conducted. All data are presented as mean ± standard deviation (SD). *: *p* < 0.05; **: *p* < 0.01.

## 3. Results

### 3.1. STEAP3 Suppresses PRRSV Replication

**Figure 1:**
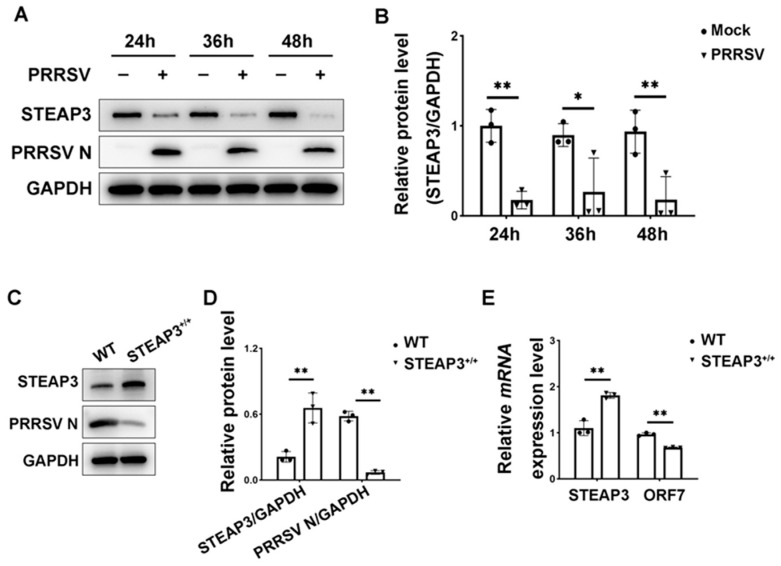
STEAP3 inhibits PRRSV replication. (**A**) and (**B**) show the expression of STEAP3 and PRRSV N protein in MARC-145 cells. Cells were infected at different time points of PRRSV (MOI = 1). (**C**) and (**D**) show the expression of STEAP3 and PRRSV N protein in MARC-145 cells. Cells were infected with PRRSV (MOI = 1) after being electroporated with pcDNA3.1-STEAP3 or the empty pcDNA3.1 vector. (**E**) Expression of *STEAP3* and PRRSV *ORF7* mRNA in MARC-145 cells. * *p* < 0.05; ** *p* < 0.01; two-way ANOVA test; n = 3.

To verify the relationship between STEAP3 and PRRSV, we conducted experiments using MARC-145 cells infected with PRRSV at various time intervals. The findings revealed that the protein expression of STEAP3 notably declined following PRRSV infection, with this reduction being dependent on the time elapsed (Figure 1A,B). Additionally, when STEAP3 was overexpressed, there was a significant decrease in the expression levels of PRRSV *ORF7* mRNA and N protein (Figure 1C–E). This suggests that STEAP3 plays a crucial role in inhibiting PRRSV replication.

### 3.2. Transcriptomics Sequencing and Analysis

**Figure 2:**
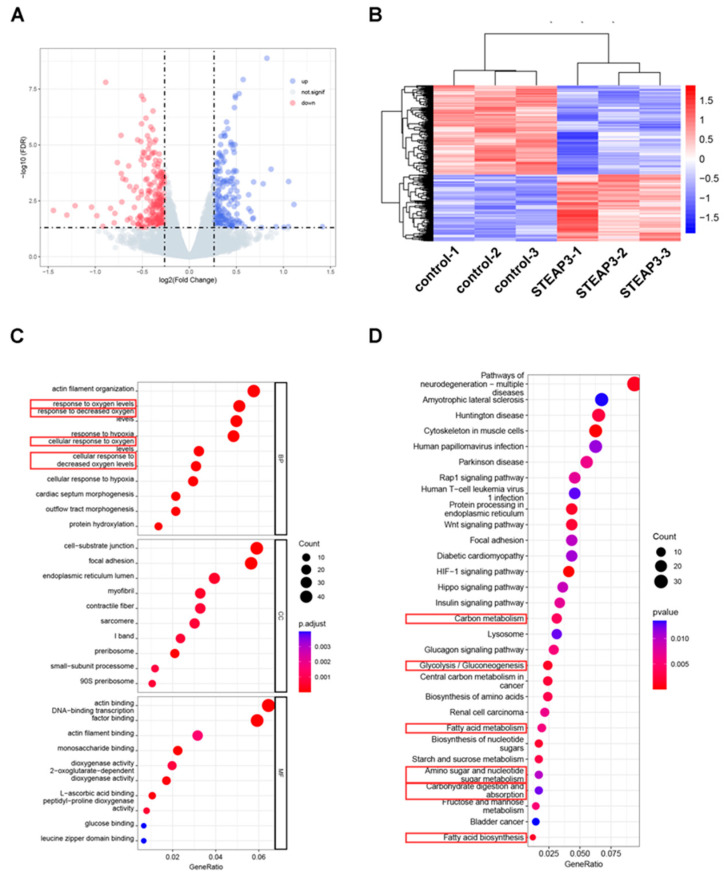
The impact of STEAP3 on the transcriptomic profile in MARC-145 cells with PRRSV infection. (**A**) The volcano plot of DEGs. (**B**) The heat map of DEGs. (**C**) Gene Ontology (GO) and (**D**) Kyoto Encyclopedia of Genes and Genomes (KEGG) pathways analysis of DEGs. Red box: GO and KEGG pathways related to lipid metabolism.

To explore the mechanism by which STEAP3 inhibits PRRSV replication, MARC-145 cells that overexpress STEAP3 were infected with PRRSV and subsequently underwent transcriptome sequencing. The differential expression analysis revealed a total of 400 differentially expressed genes (DEGs) (fold change > 1.5 and *p* < 0.05), with 176 DEGs showing increased expression and 224 DEGs showing decreased expression (Figure 2A,B). The functional enrichment analysis of differentially expressed genes indicated significant enrichment in terms of the response to oxygen levels, response to decreased oxygen levels, cellular response to oxygen levels, and cellular response to decreased oxygen levels in biological processes (BP) (Figure 2C). In KEGG pathways, significant enrichment was observed in fatty acid biosynthesis, fatty acid metabolism, glycolysis/gluconeogenesis, amino sugar and nucleotide sugar metabolism, carbohydrate digestion and absorption, and carbon metabolism (Figure 2D). These findings suggest that STEAP3 may inhibit PRRSV replication by influencing fatty acid synthesis and energy metabolism.

### 3.3. Metabolomics and Analysis

**Figure 3:**
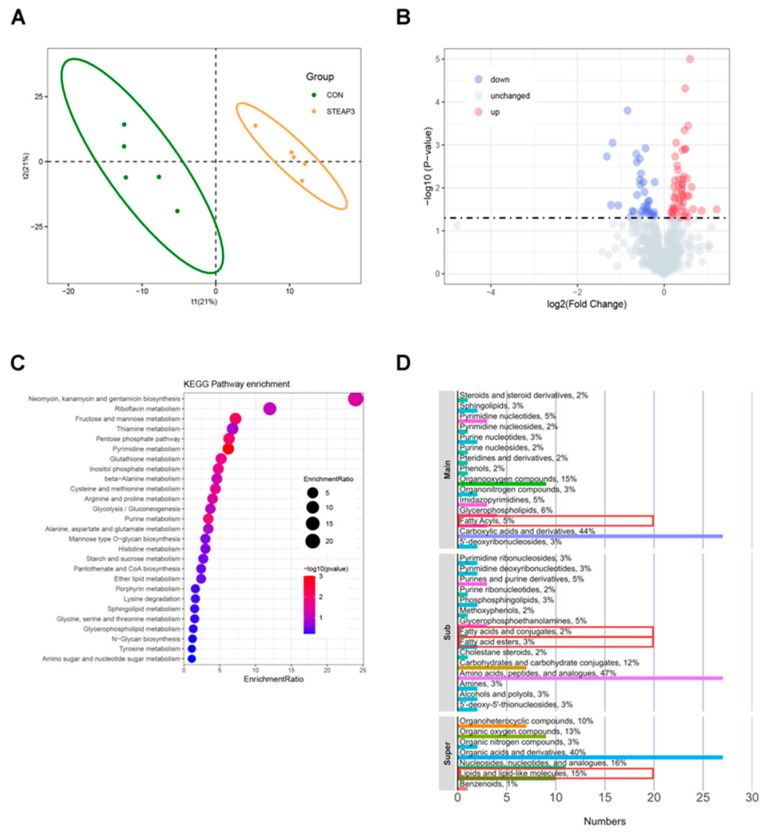
The effect of STEAP3 on metabolomics in MARC-145 cells with PRRSV infection. (**A**) The OPLS-DA plot of the STEAP3 overexpression group and the control group. (**B**) The volcano plot of DEMs. (**C**) KEGG pathways analysis of DEMs. (**D**) Chemical classification analysis of DEMs. Red box: proportion of small-molecule metabolites related to fatty acids and lipid metabolism.

Lipid synthesis and energy metabolism are accompanied by changes in small-molecule metabolites. Therefore, non-targeted metabolomics sequencing was conducted using the aforementioned materials. The results of the OPLS-DA analysis indicated that the experimental group and the treatment group were significantly separated into two regions (Figure 3A). The analysis of differentially expressed metabolites (DEMs) revealed a total of 86 DEMs (*p* < 0.05, VIP > 1.0), of which 52 were upregulated and 34 were downregulated (Figure 3B). The KEGG enrichment results showed that the DEMs were mainly enriched in pathways such as glycolysis/gluconeogenesis, ether lipid metabolism, and amino sugar and nucleotide sugar metabolism (Figure 3C). The chemical classification results of the DEMs indicated that 5% of the metabolites belonged to the fatty acyls category (main class); 2% and 3% of the metabolites were in the fatty acids and conjugates and fatty acid esters categories (sub class), respectively; and 15% of the metabolites were in the lipids and lipid-like molecules category (super class) (Figure 3D).

### 3.4. Combined Analysis of Transcriptomics and Metabolomics

**Figure 4:**
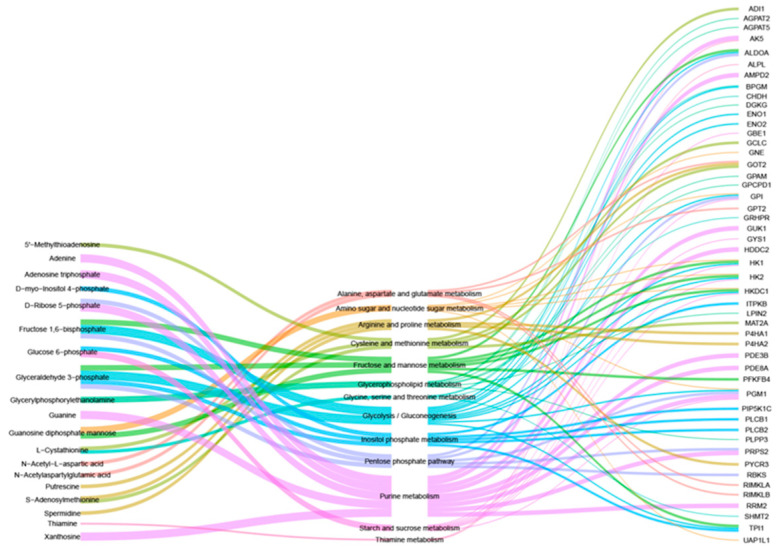
The co-effect of STEAP3 on transcriptomics and metabolomics in MARC-145 cells with PRRSV infection. Left: DEMs; middle: KEGG shared by DEMs and DEGs; right: DEGs.

To further investigate the effect of STEAP3 on lipogenesis, a combined analysis was performed using the previously mentioned transcriptomic and metabolomic results. The analysis revealed that 19 DEMs and 49 DEGs were linked to 13 KEGG pathways. Notably, amino sugar and nucleotide sugar metabolism, glycolysis/gluconeogenesis, and glycerophospholipid metabolism are significant contributors to lipogenesis and energy metabolism (Figure 4). Consequently, STEAP3 may hinder PRRSV replication by influencing lipogenesis and energy metabolism.

### 3.5. STEAP3 Inhibits FASN Expression and Promotes LD Synthesis

**Figure 5:**
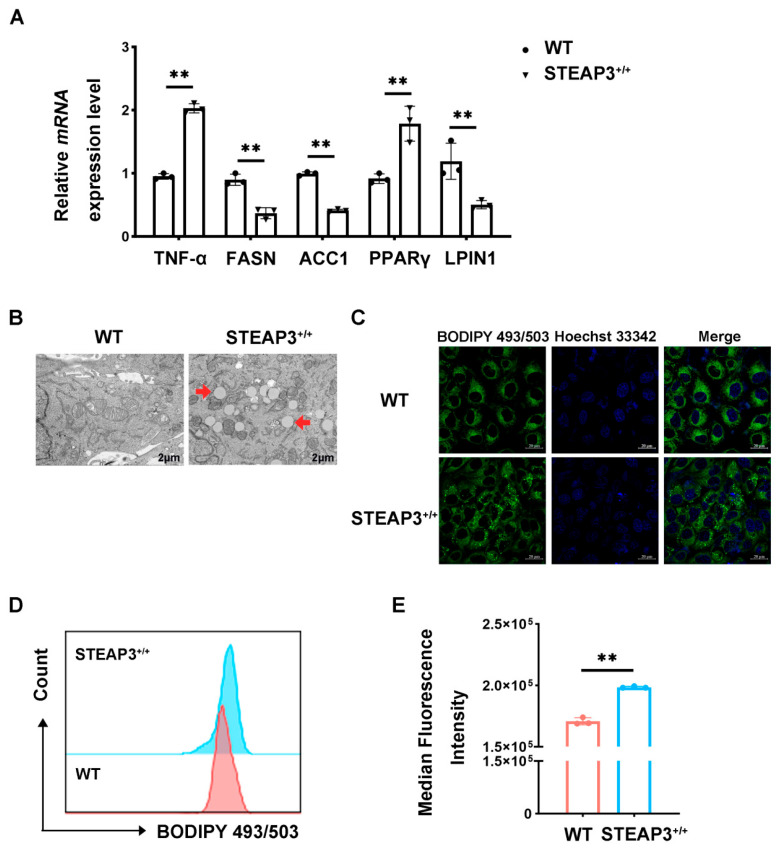
STEAP3 inhibits FASN expression and promotes LD synthesis. (**A**) Expression of *FASN*, *ACC1*, *PLIN1*, *PPARγ*, and *TNF-α* mRNA in MARC-145 cells. Cells were infected with PRRSV (MOI = 1) after being electroporated with pcDNA3.1-STEAP3 and the empty pcDNA3.1 vector. (**B**) Representative TEM images of MARC-145 cells. The cells’ treatment was the same as above. Scale bars: 2 μm. (**C**) Confocal images of PRRSV-infected MARC-145 cells with or without STEAP3 overexpression for 24 h. BODIPY^+^ puncta represented LDs. Scale bars: 20 μm. (**D**) and (**E**) show the cytometry analysis of LDs’ fluorescence intensity in MARC-145 cells. The cells’ treatment was the same as above. ** *p* < 0.01; two-way ANOVA test; n ≥ 3.

This study, based on the combined analysis results, examines lipogenesis and assesses the effects of STEAP3 overexpression on important genes related to this process. The quantitative real-time PCR findings revealed that STEAP3 overexpression notably reduced the mRNA levels of *FASN*, *ACC1*, and *PLIN1* while increasing the mRNA levels of *PPARγ* and *TNF-α* (Figure 5A). Furthermore, the transmission electron microscopy results showed that STEAP3 overexpression facilitated the development of intracellular LDs (Figure 5B). In addition, we also observed that the overexpression of STEAP3 in PRRSV-infected MARC-145 cells enhanced the staining of LDs (Figure 5C). The flow analysis results indicated that STEAP3 overexpression significantly increased the fluorescence intensity of intracellular LDs (Figure 5D,E), suggesting that STEAP3 effectively enhances LD synthesis. These findings imply that STEAP3 could inhibit FASN expression and alter LD synthesis, thereby reducing PRRSV replication.

## 4. Discussion

Viral infections can significantly impact various essential cellular functions and metabolic processes, including autophagy, apoptosis, mitochondrial activity, glucose metabolism, and lipid metabolism [19,20]. Lipids, which are vital components of cell membranes and serve as energy reserves, are crucial for the invasion and replication of RNA viruses [21]. Lipid droplets (LDs), which are organelles in the cytoplasm that store energy and are rich in neutral lipids, can interact with the endoplasmic reticulum, mitochondria, and peroxisomes [22,23]. These LDs release fatty acids by breaking down neutral lipids and transporting them to mitochondria or peroxisomes for β-oxidation, playing a role in the infection and replication of various RNA viruses [24]. Research has indicated that the structural proteins of DENV bind to LDs in a potassium ion-dependent manner, inhibiting viral replication [25]. Additionally, DENV can reduce the accumulation of intracellular LDs through autophagy (lipophagy), which aids in its replication [9]. In our study, we observed that PRRSV infection leads to a time-dependent decrease in STEAP3 expression, and that overexpressing STEAP3 can inhibit viral replication. Transmission electron microscopy revealed that the overexpression of STEAP3 significantly enhances LDs’ formation in MARC-145 cells infected with PRRSV, highlighting the importance of LDs in PRRSV replication. Previous research has shown that following PRRSV infection, host cells modulate the synthesis of intracellular LDs via YY1, which contributes to antiviral activity [3]. Additionally, EGCG has been found to inhibit PRRSV replication by interfering with lipid metabolism, while PRRSV activates lipid synthesis through the ROS-dependent AKT/PCK1/INSIG/SREBPs pathway [4,26]. This indicates that lipid or lipid droplet synthesis is crucial for PRRSV replication, offering new avenues for anti-PRRS research.

STEAP3 is recognized as a marker of ferroptosis, with lipid peroxidation being its most significant characteristic. Previous research using iron-bound red blood cells has shown that while STEAP3 appears to enhance reductase activity, it also seems to paradoxically increase oxidative damage [27]. However, fatty acids need oxidation for energy production. Studies related to STEAP, including those on non-alcoholic fatty liver disease, cardiac hypertrophy, and insulin resistance, suggest its involvement in lipid metabolism [13,14,17]. This study utilized transcriptomics and metabolomics to reveal that STEAP3 can significantly influence fatty acid synthesis, lipogenesis, and various oxidative processes during PRRSV infection. Furthermore, the QPCR results indicated that STEAP3 notably downregulated the expression of lipogenesis-related genes such as ACC1, FASN, and PLIN1 while significantly upregulating peroxisome proliferator-activated receptor gamma (PPARγ). These findings suggest that STEAP3 plays a crucial role in regulating lipid metabolism. Additionally, the observation that STEAP3 can significantly increase TNF-α levels and inhibit PRRSV replication implies that during PRRSV infection, STEAP3 may negatively regulate fatty acid synthesis, positively regulate PPARγ, promote LDs’ formation, and suppress PRRSV replication. Previous studies have demonstrated that the host gene YY1 can suppress FASN expression and reduce fatty acid synthesis while simultaneously promoting PPARγ expression to increase LD synthesis, which in turn hinders PRRSV replication [3]. Additionally, the host gene STEAP3 is capable of modulating intracellular inflammatory responses [28]. These results align with our findings. This presents a new target for PRRS prevention and control. The results indicate that managing LDs’ generation or lipid metabolism could serve as an effective antiviral treatment strategy. Previous studies have demonstrated that Pep14-23, a peptide that targets the DENV capsid, disrupts the interaction between the capsid and LDs, thereby reducing DENV replication [29]. Additionally, triacsin C, an ACSL inhibitor, has proven itself to be highly effective in limiting rotavirus (RV) replication [30].

## 5. Conclusions

This research demonstrates that the upregulation of STEAP3 plays a significant role in modulating lipid metabolism and the formation of LDs upon PRRSV infection. The underlying mechanism appears to involve the effect of STEAP3 on the expression of several regulatory genes and metabolites associated with lipid metabolism, which in turn affects LDs’ formation and ultimately inhibits the replication of PRRSV. Our findings contribute novel insights and potential targets regarding the function of STEAP3 in the regulatory mechanisms of PRRSV replication, thereby providing a theoretical foundation for the development of innovative strategies aimed at preventing and controlling PRRS through the modulation of lipid metabolism.

## Figures and Tables

**Table 1:** List of primers used in the quantitative real-time PCR analysis.

Primers	Primer Sequences (5′→3′)
ORF7 (N)-F	AAAACCAGTCCAGAGGCAAG
ORF7 (N)-R	CGGATCAGACGCACAGTATG
STEAP3-F	CCAATGCTGAGTACCTGGC
STEAP3-R	ATCTCCGAGACAGCACGC
FASN-qF	CACATCGTTCGAGCAGCATG
FASN-qR	AATTTCCAGGAAGCGACCGT
ACC1-qF	TAGTCTGCCACGGATCCAGA
ACC1-qR	GGGAGGGATCTCTGAGGGTT
PPARγ-qF	TGAGTTCGCTGTGAAGTT
PPARγ-qR	CAATCTGTCTGAGGTCTGT
TNF-α-qF	CTGTAGGTTGCTCCCACCTG
TNF-α-qR	CCAGTAGGGCGGTTACAGAC
LPIN1-qF	CAAGCAAGTAGGAGTGTCT
LPIN1-qR	GCGGAGGCAGAATGAATA
GAPDH-F	TGACAACAGCCTCAAGATCG
GAPDH-R	GTCTTCTGGGTGGCAGTGAT

## Data Availability

The datasets presented in this study can be found in online repositories. The names of the repository/repositories and accession number(s) can be found below: https://www.ncbi.nlm.nih.gov/, NO. PRJNA1208659.

## Supplementary Materials

The following supporting information can be downloaded at: https://www.mdpi.com/article/10.3390/vetsci12020147/s1.
