# Supplementary figures and images for: STEAP3 Inhibits Porcine Reproductive and Respiratory Syndrome Virus Replication by Regulating Fatty Acid and Lipid Droplet Synthesis
Source: Vet Sci. 2025 Feb 8;12(2):147. doi: 10.3390/vetsci12020147 (PMC11861627; doi:10.3390/vetsci12020147)

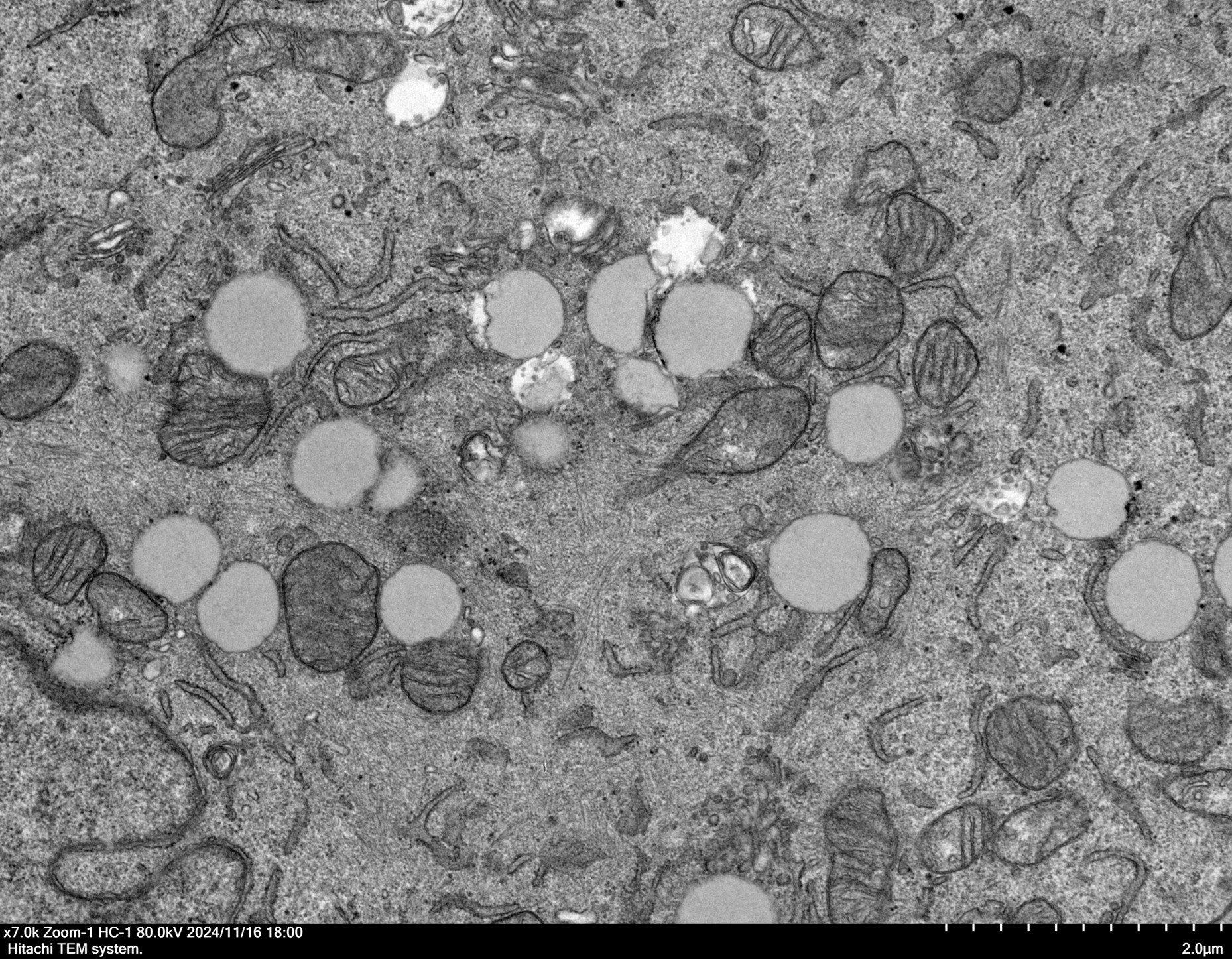

Supplement: Supplementary file 1 [file vetsci-12-00147-s001.zip › raw data/fig5/B-Transmission Electron Microscope (TEM)/STEAP3/main/图片1.jpg]

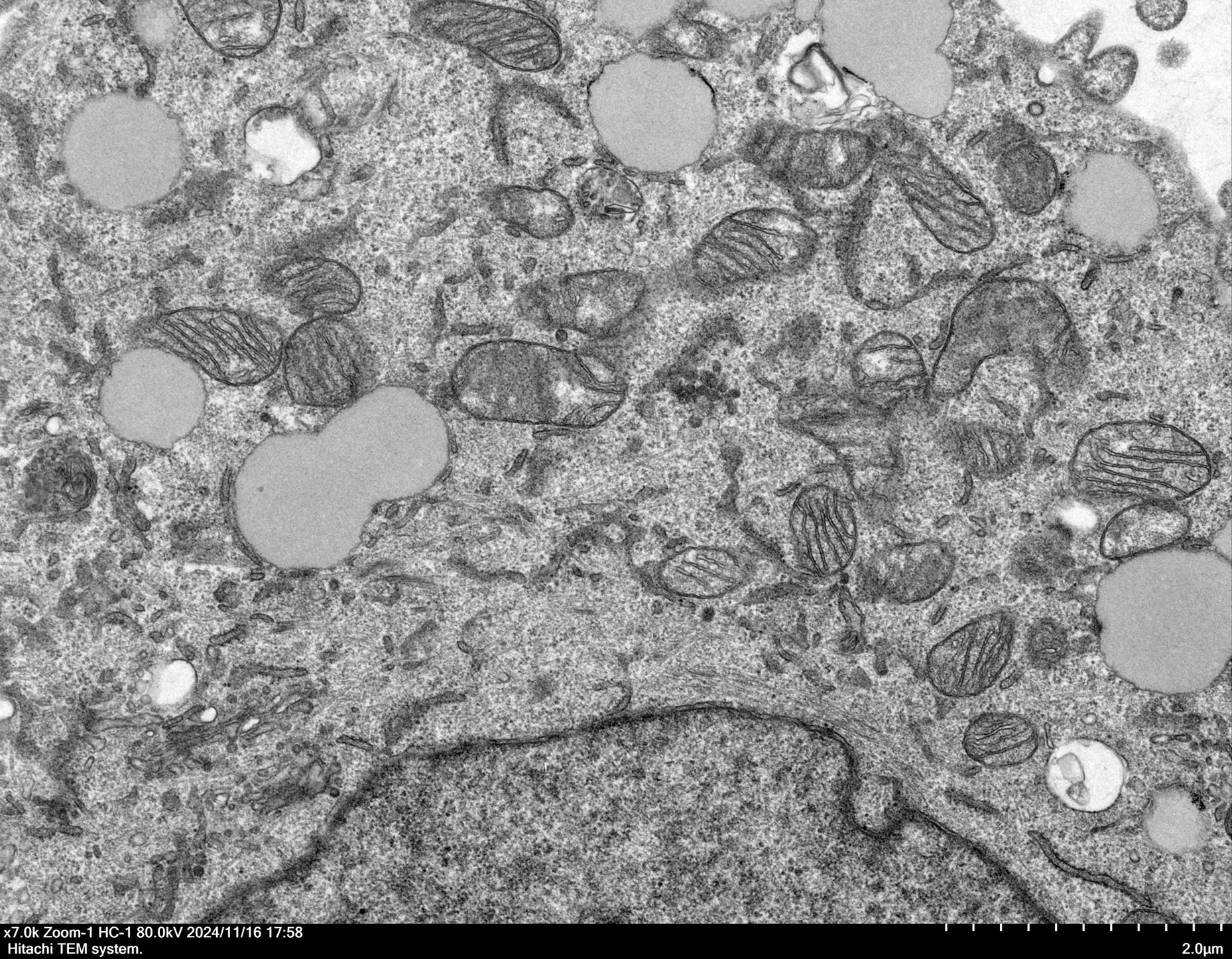

Supplement: Supplementary file 1 [file vetsci-12-00147-s001.zip › raw data/fig5/B-Transmission Electron Microscope (TEM)/STEAP3/repeats/图片2.jpg]

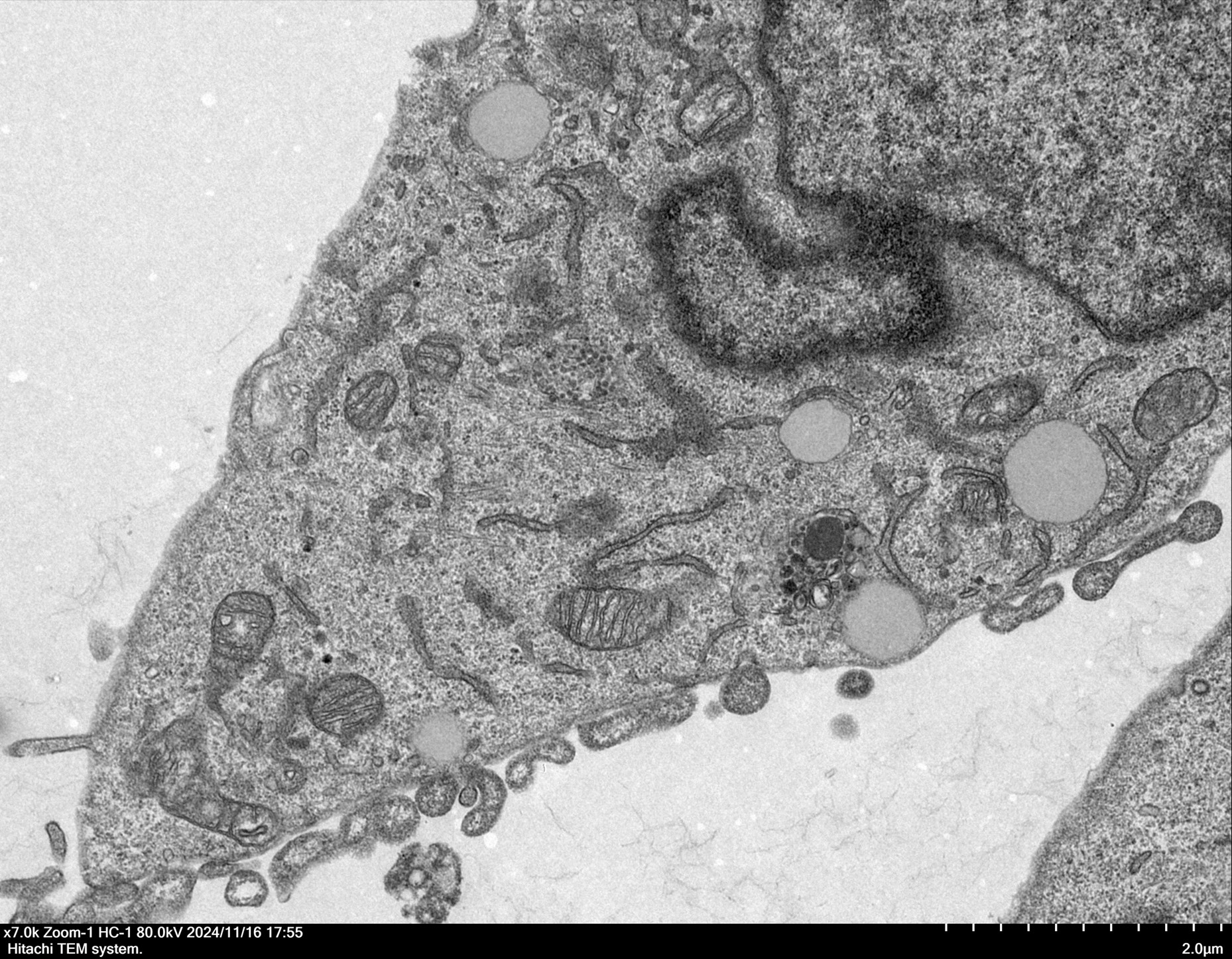

Supplement: Supplementary file 1 [file vetsci-12-00147-s001.zip › raw data/fig5/B-Transmission Electron Microscope (TEM)/STEAP3/repeats/图片3.jpg]

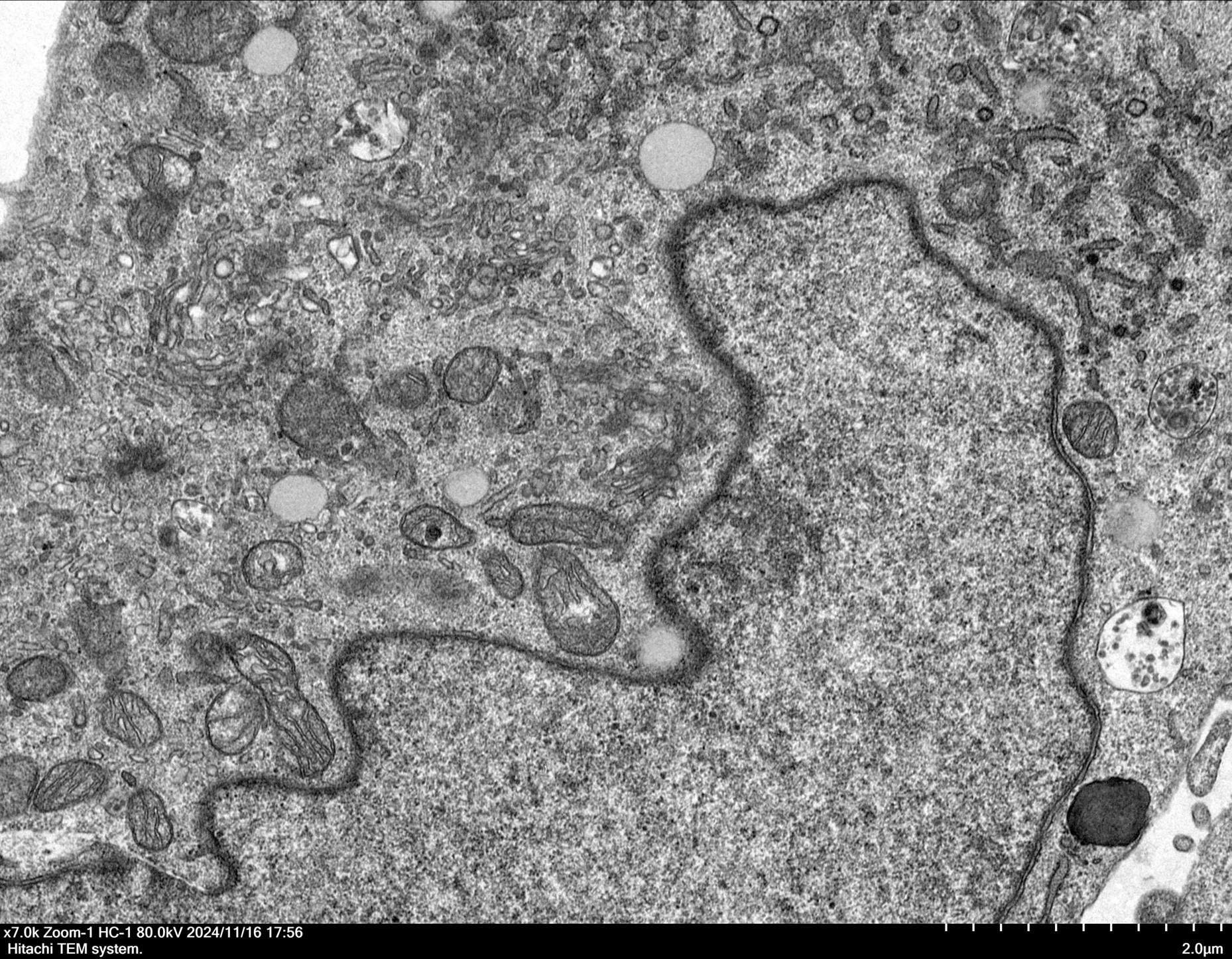

Supplement: Supplementary file 1 [file vetsci-12-00147-s001.zip › raw data/fig5/B-Transmission Electron Microscope (TEM)/STEAP3/repeats/图片4.jpg]

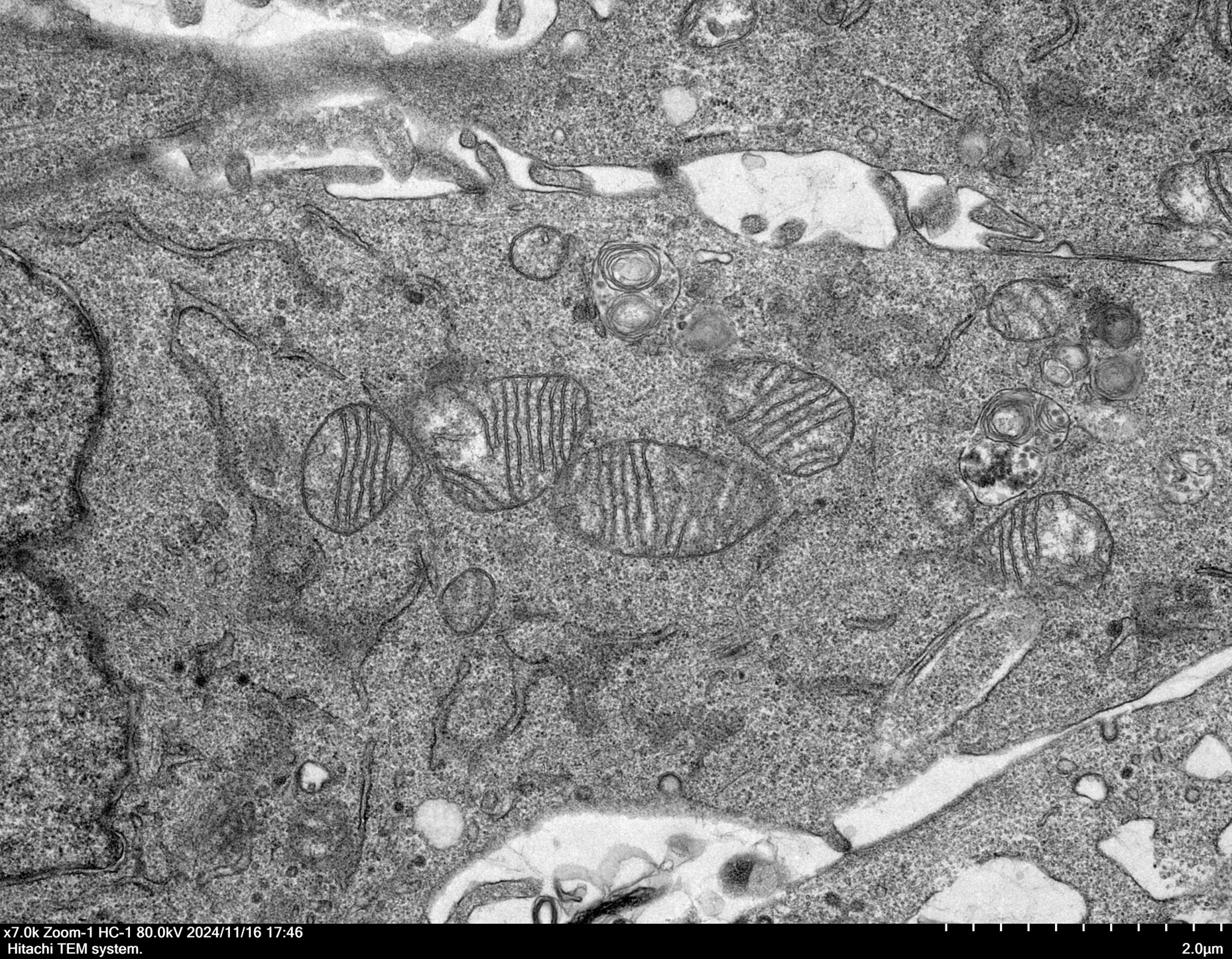

Supplement: Supplementary file 1 [file vetsci-12-00147-s001.zip › raw data/fig5/B-Transmission Electron Microscope (TEM)/WT/main/图片1.jpg]

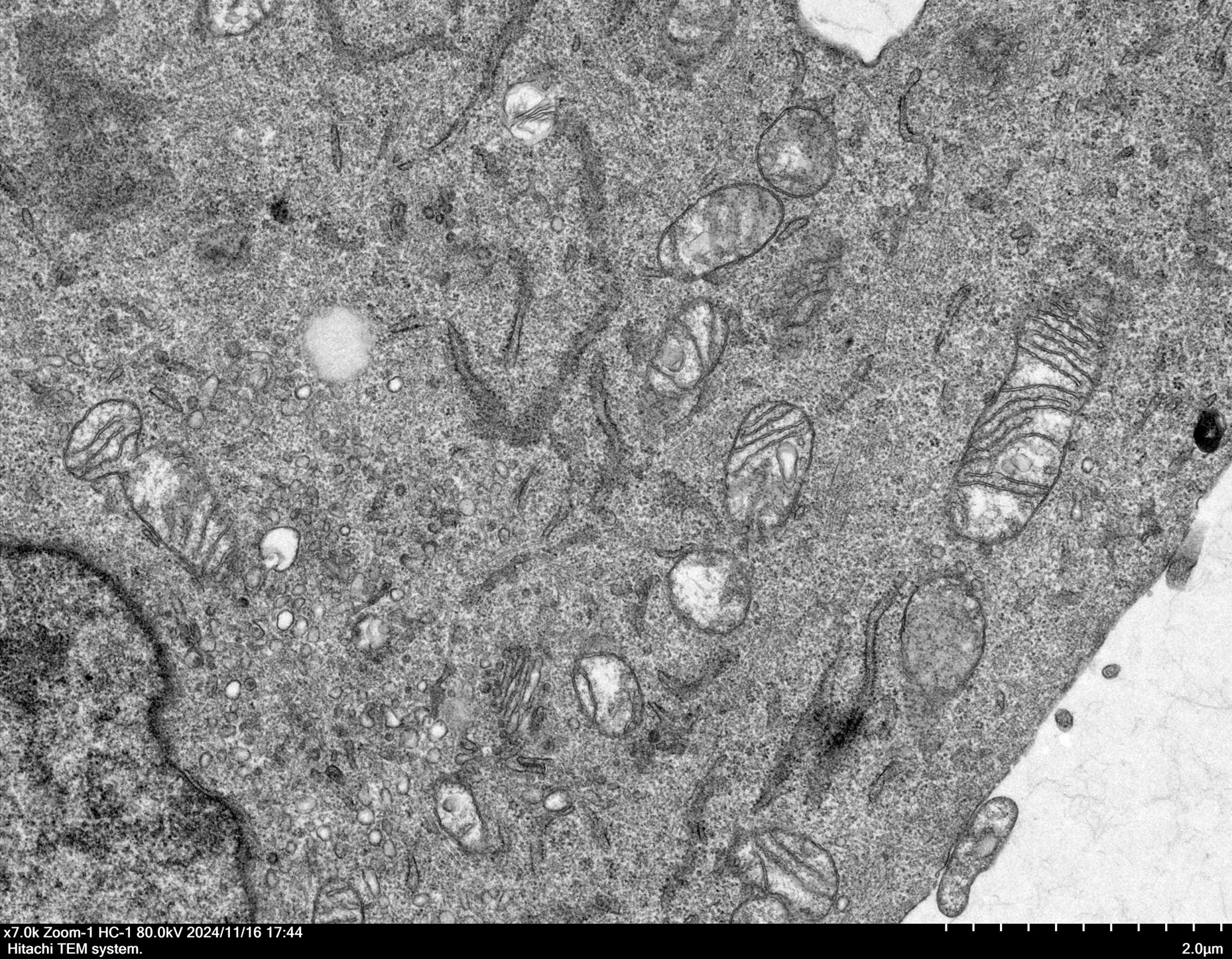

Supplement: Supplementary file 1 [file vetsci-12-00147-s001.zip › raw data/fig5/B-Transmission Electron Microscope (TEM)/WT/repeats/图片2.jpg]

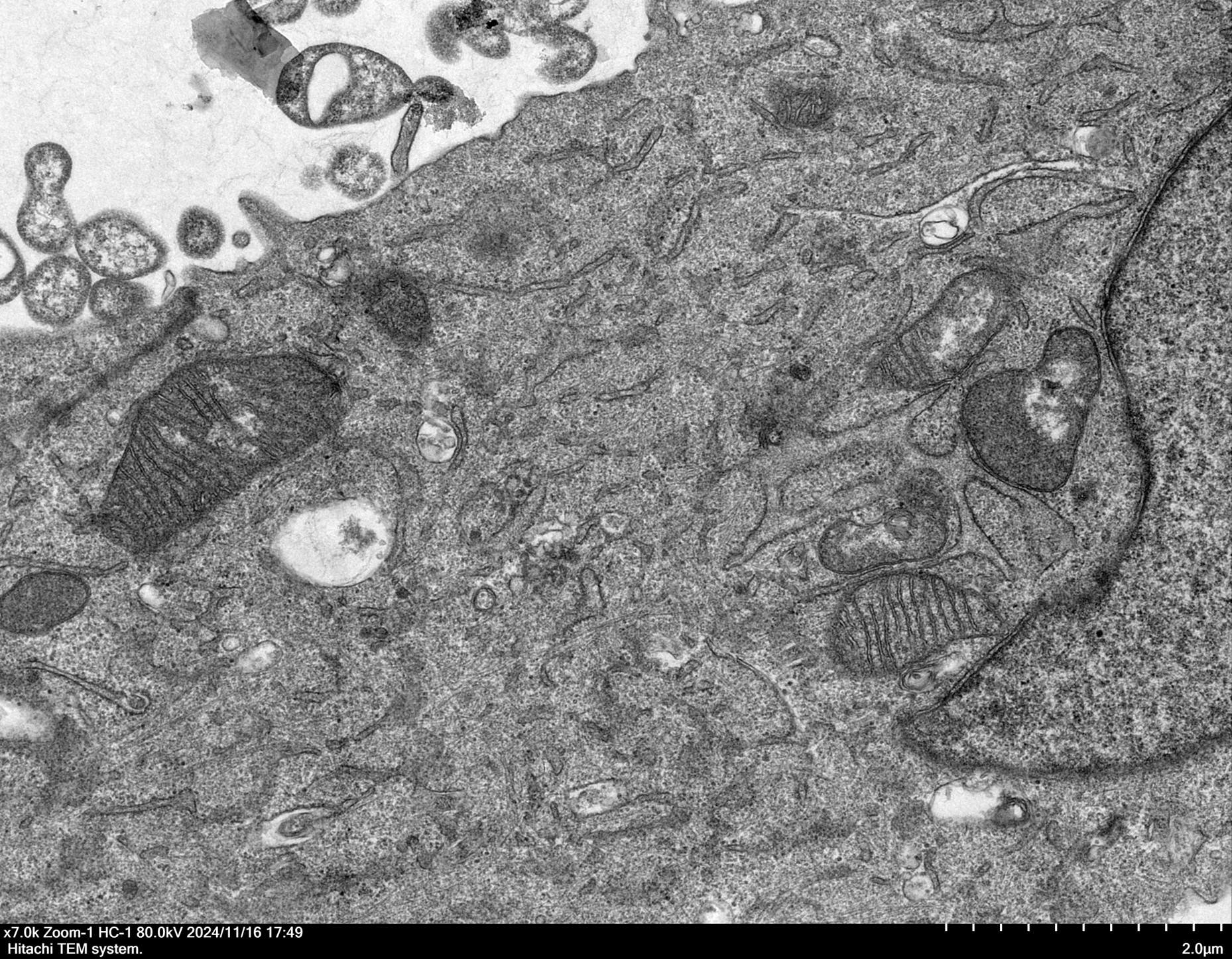

Supplement: Supplementary file 1 [file vetsci-12-00147-s001.zip › raw data/fig5/B-Transmission Electron Microscope (TEM)/WT/repeats/图片3.jpg]

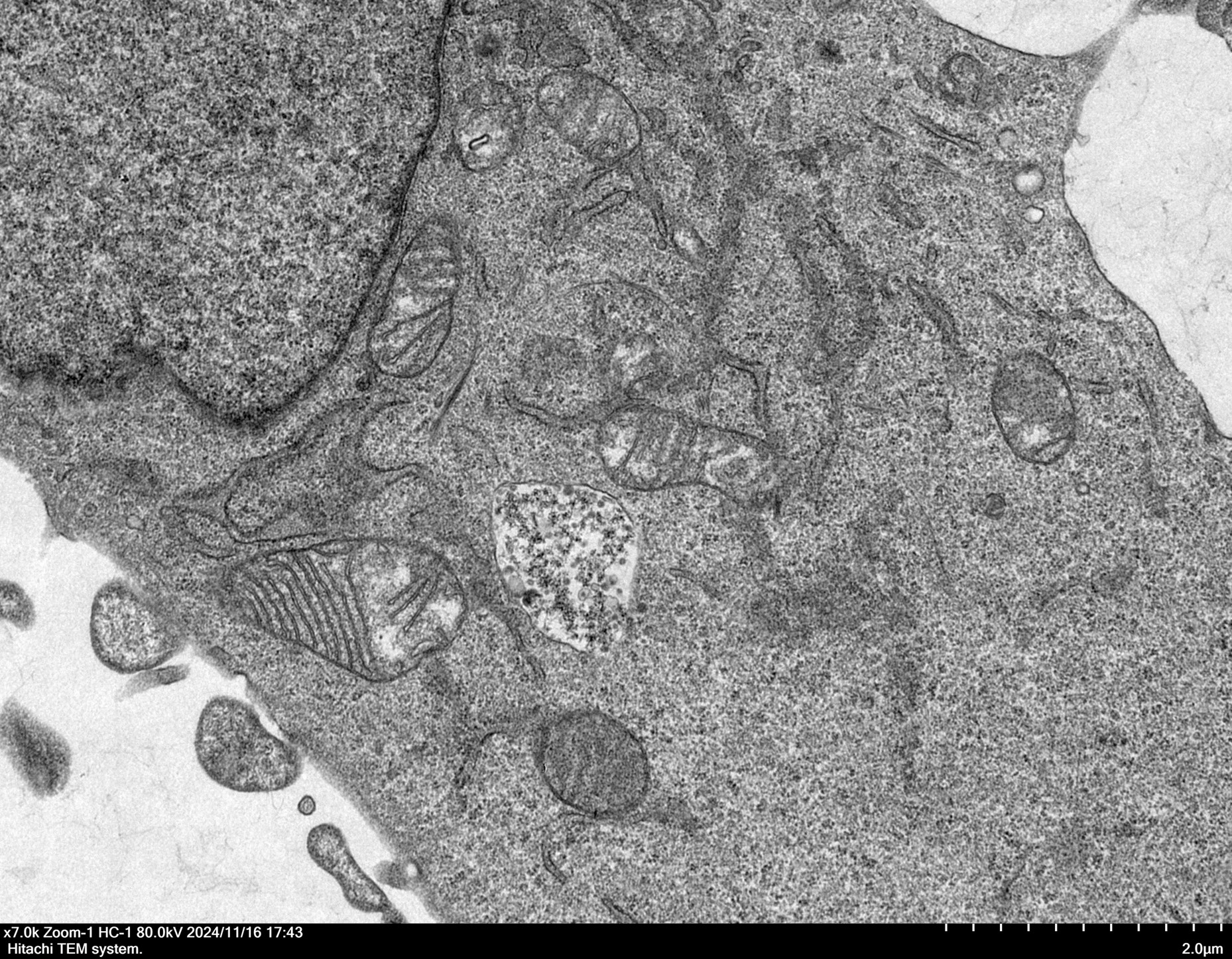

Supplement: Supplementary file 1 [file vetsci-12-00147-s001.zip › raw data/fig5/B-Transmission Electron Microscope (TEM)/WT/repeats/图片4.jpg]

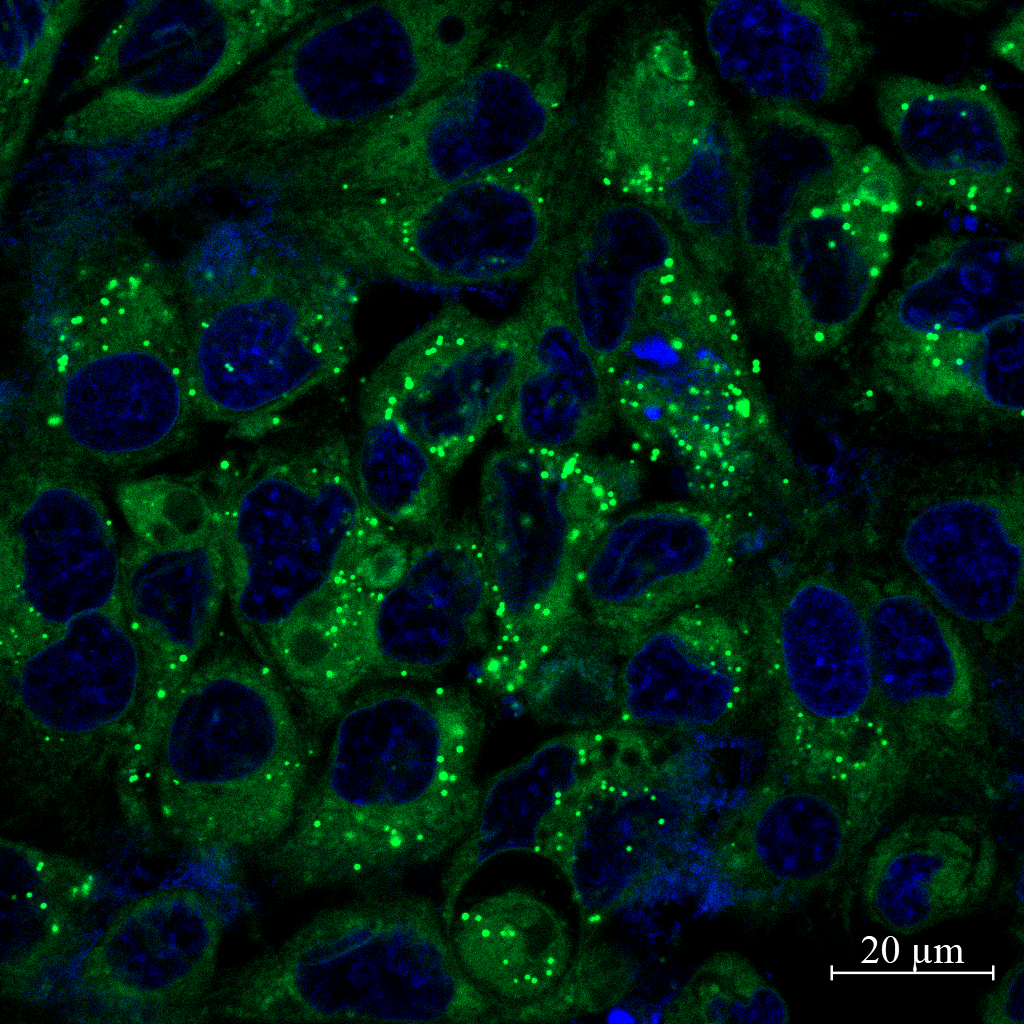

Supplement: Supplementary file 1 [file vetsci-12-00147-s001.zip › raw data/fig5/C-Microscopy/main/STEAP3-1/STEAP3-1.tif]

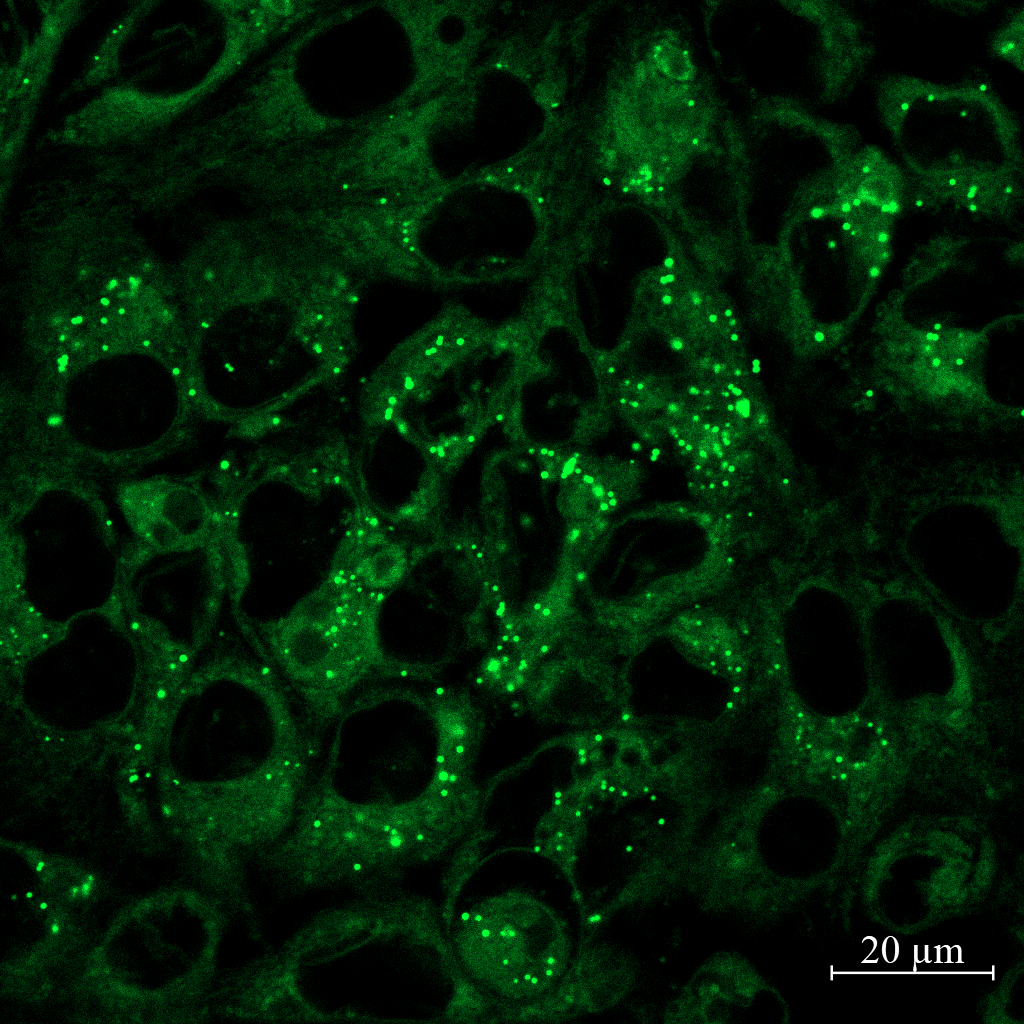

Supplement: Supplementary file 1 [file vetsci-12-00147-s001.zip › raw data/fig5/C-Microscopy/main/STEAP3-1/STEAP3-1_AF488-T1.tif]

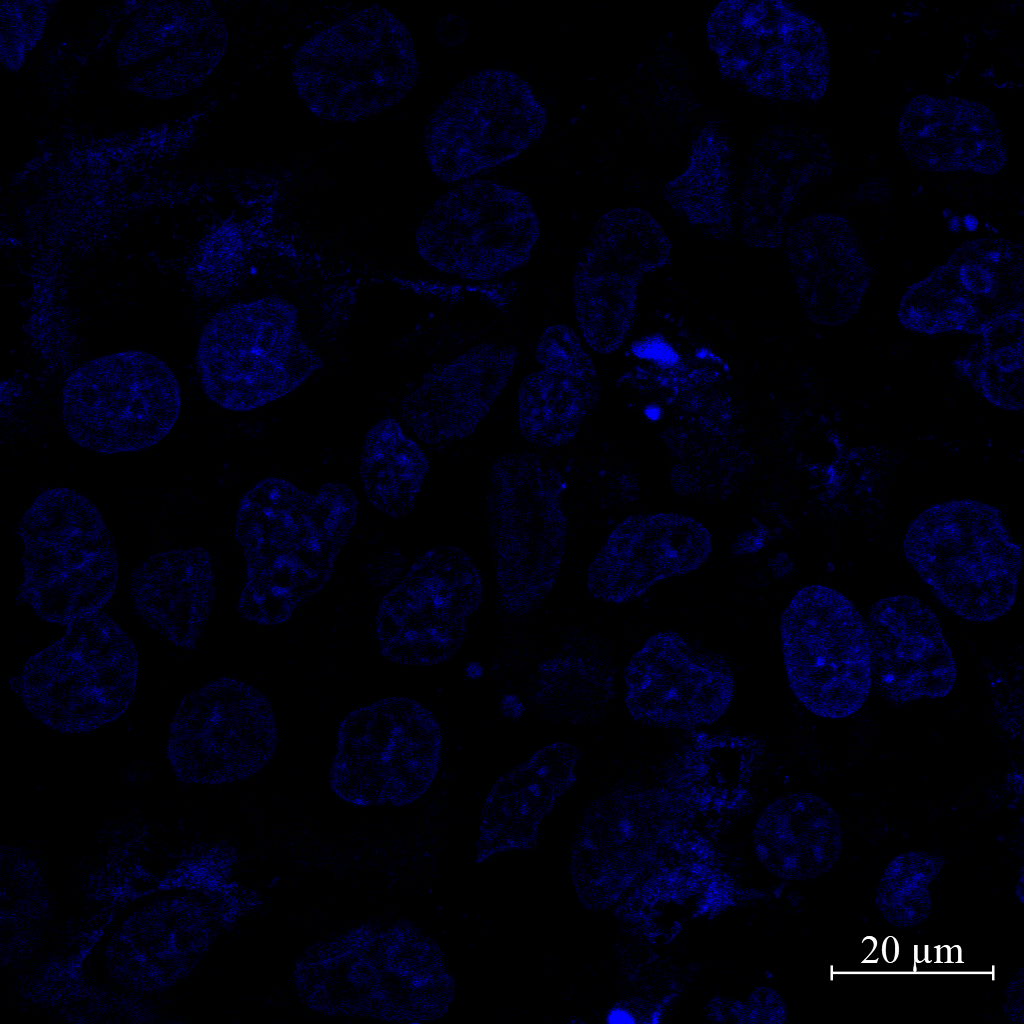

Supplement: Supplementary file 1 [file vetsci-12-00147-s001.zip › raw data/fig5/C-Microscopy/main/STEAP3-1/STEAP3-1_DAPI-T2.tif]

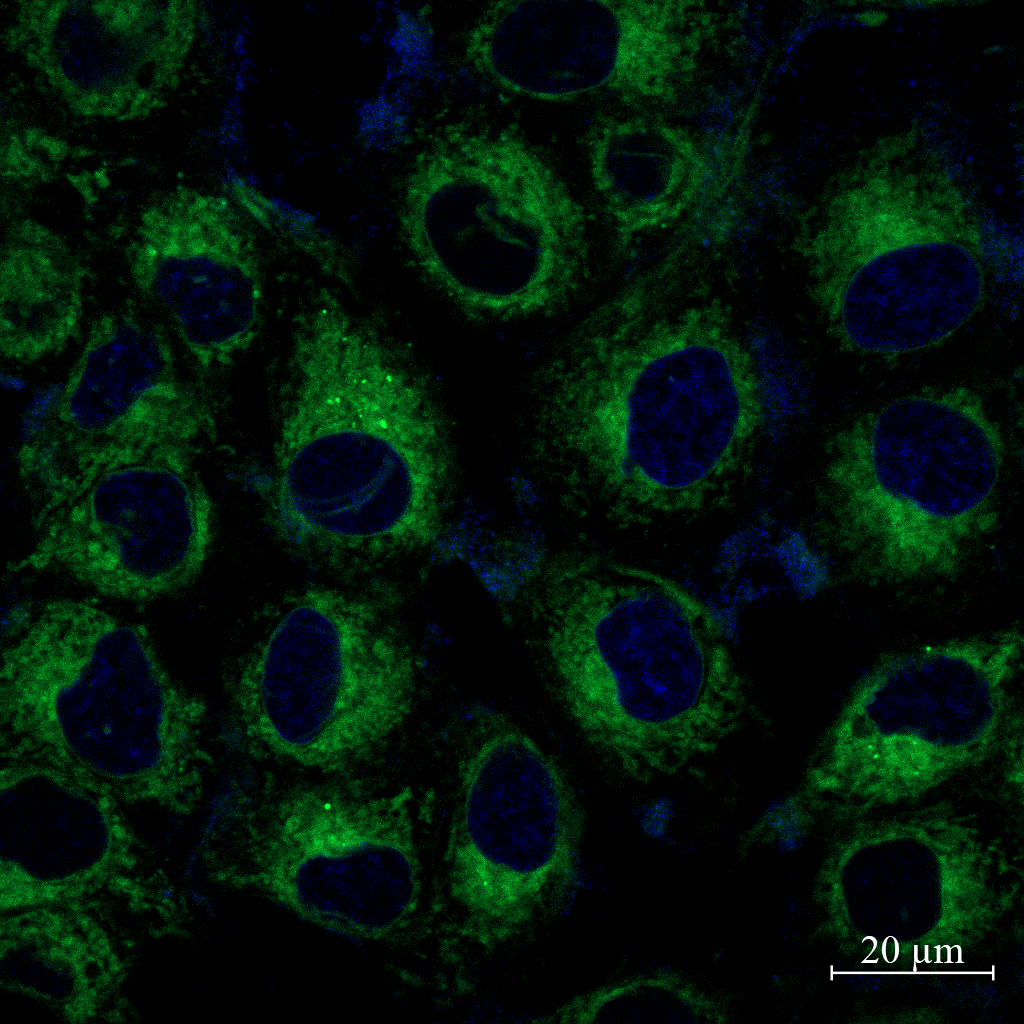

Supplement: Supplementary file 1 [file vetsci-12-00147-s001.zip › raw data/fig5/C-Microscopy/main/WT-1/WT-1.tif]

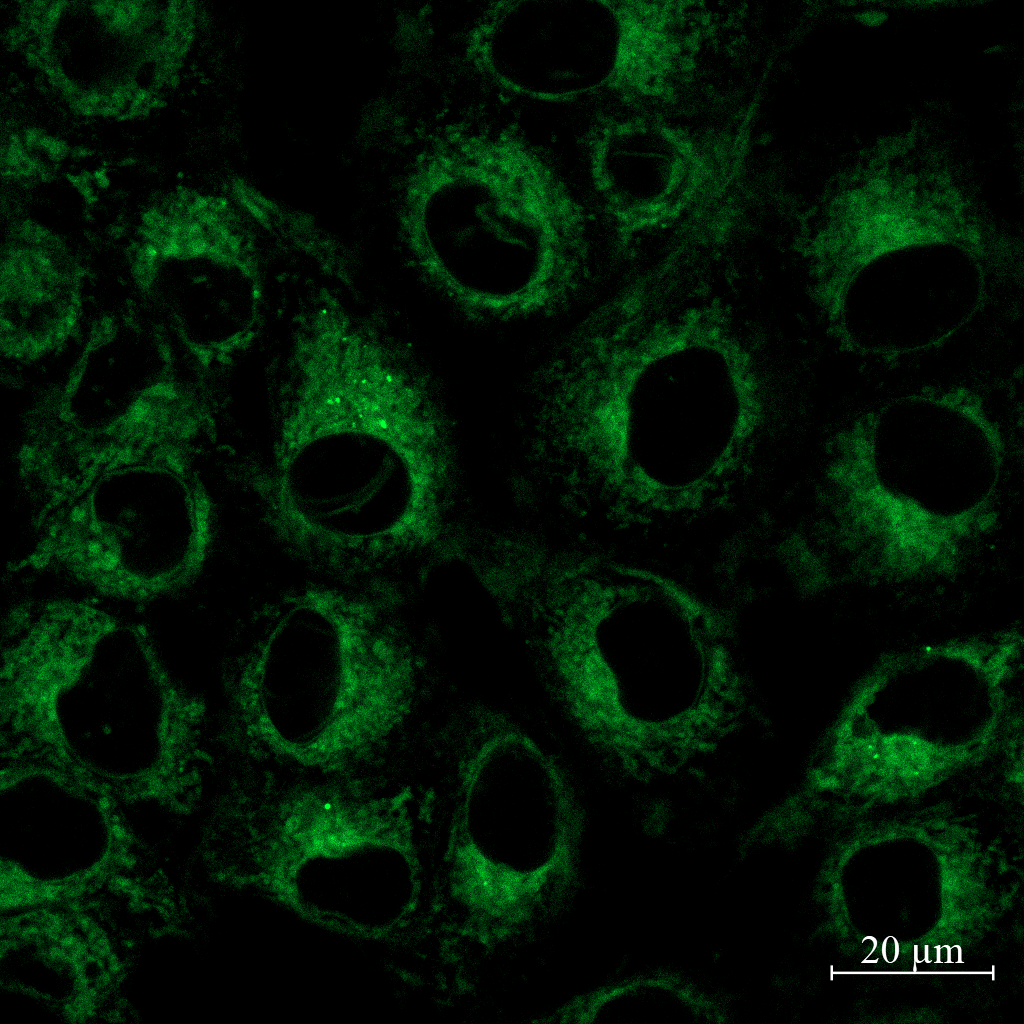

Supplement: Supplementary file 1 [file vetsci-12-00147-s001.zip › raw data/fig5/C-Microscopy/main/WT-1/WT-1_AF488-T1.tif]

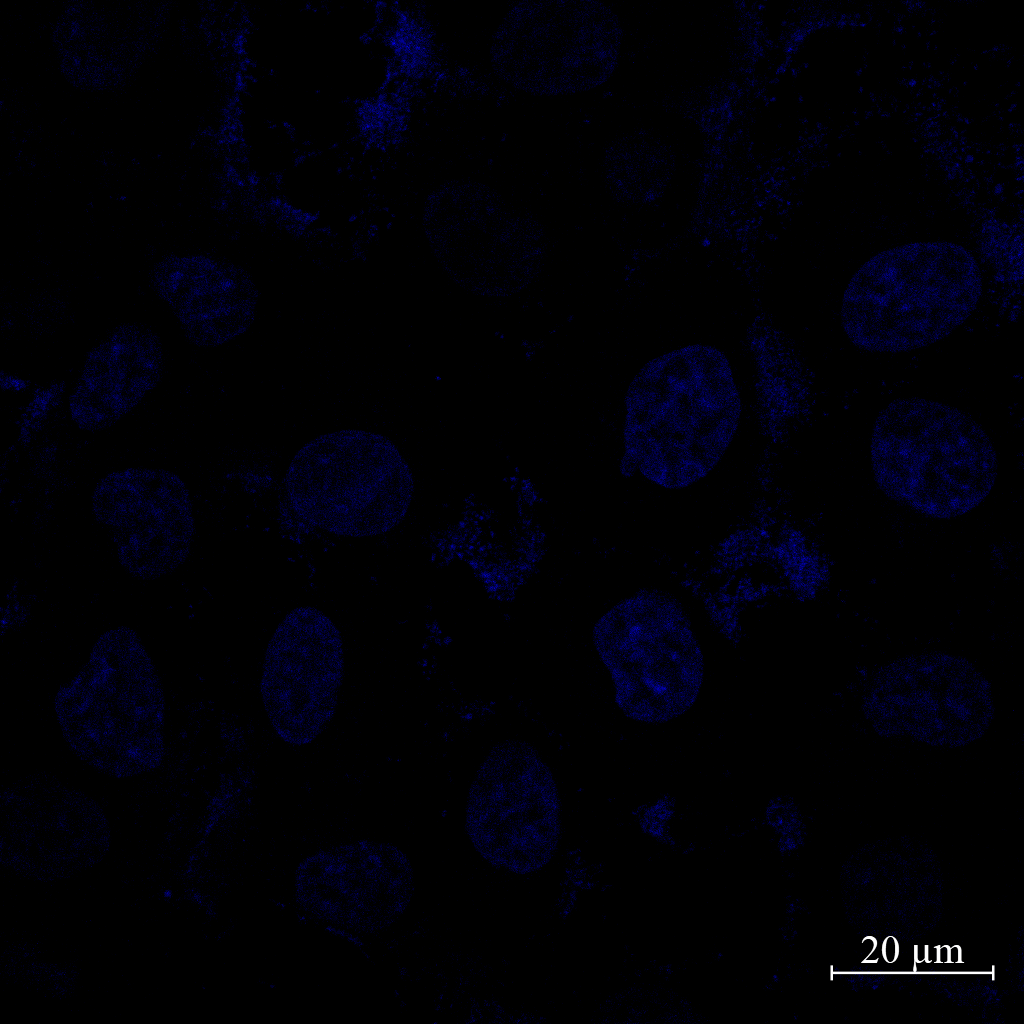

Supplement: Supplementary file 1 [file vetsci-12-00147-s001.zip › raw data/fig5/C-Microscopy/main/WT-1/WT-1_DAPI-T2.tif]

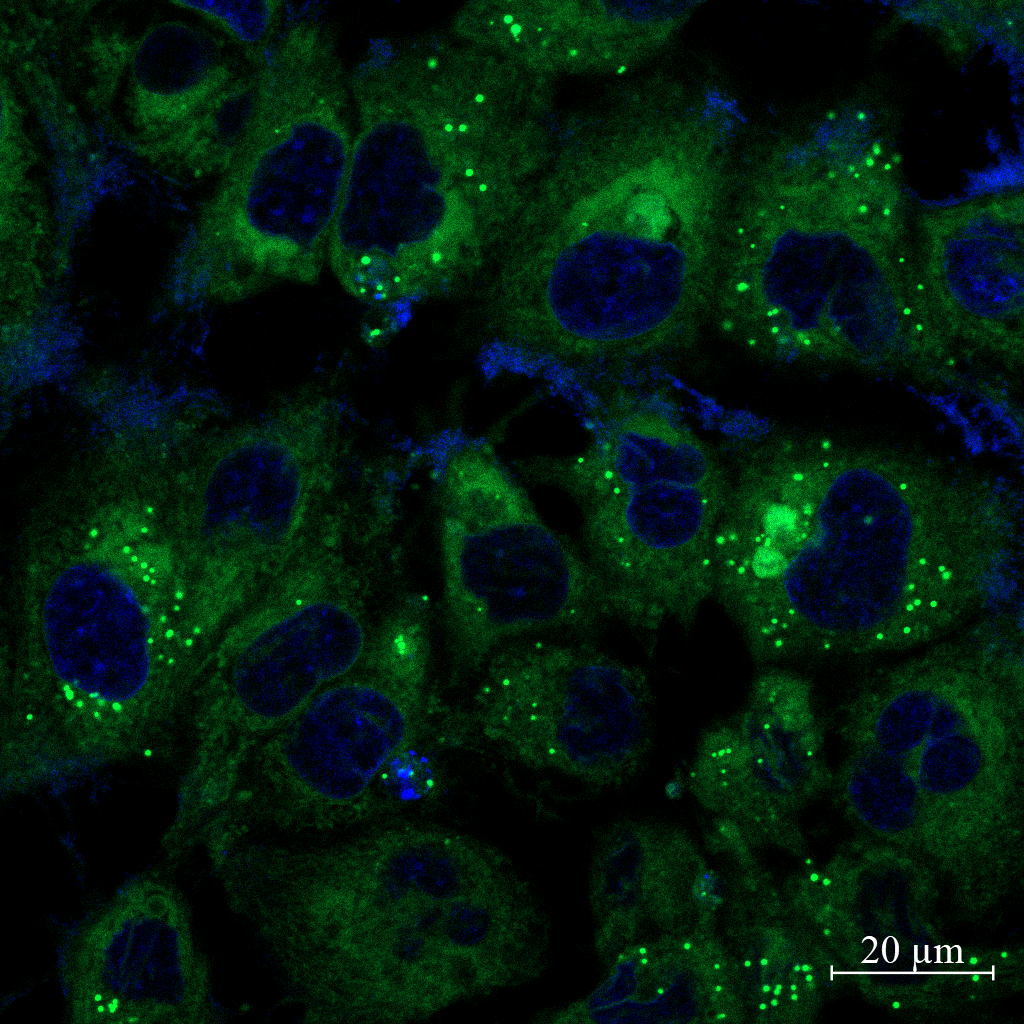

Supplement: Supplementary file 1 [file vetsci-12-00147-s001.zip › raw data/fig5/C-Microscopy/repeat 1/STEAP3-2/STEAP3-2.tif]

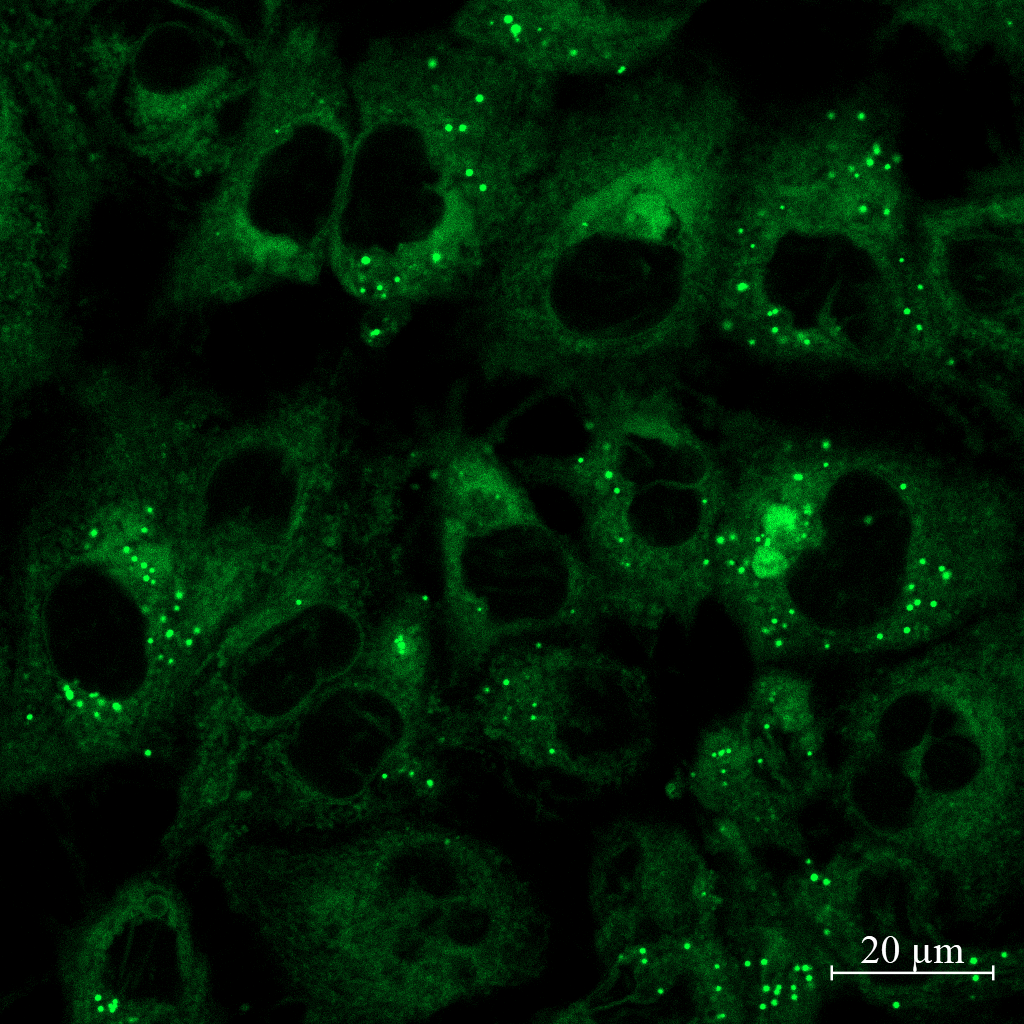

Supplement: Supplementary file 1 [file vetsci-12-00147-s001.zip › raw data/fig5/C-Microscopy/repeat 1/STEAP3-2/STEAP3-2_AF488-T1.tif]

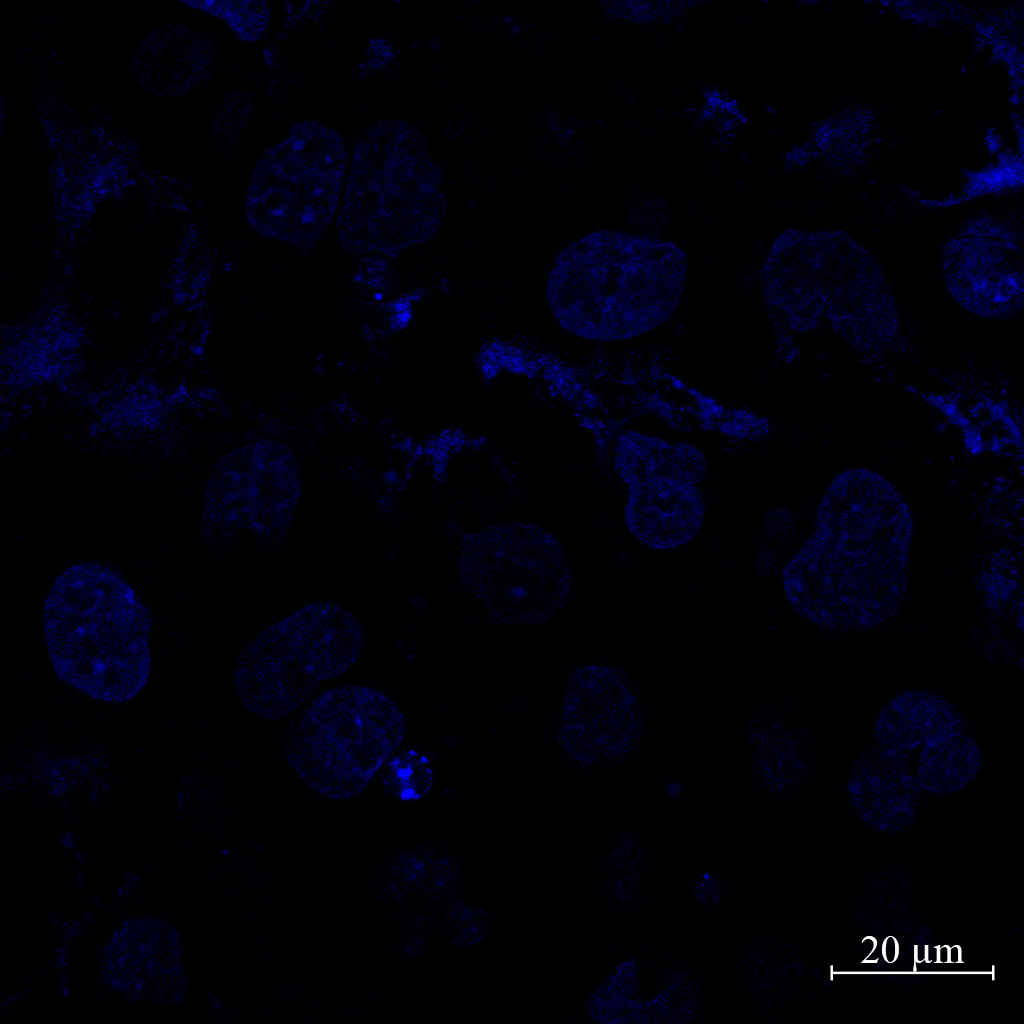

Supplement: Supplementary file 1 [file vetsci-12-00147-s001.zip › raw data/fig5/C-Microscopy/repeat 1/STEAP3-2/STEAP3-2_DAPI-T2.tif]

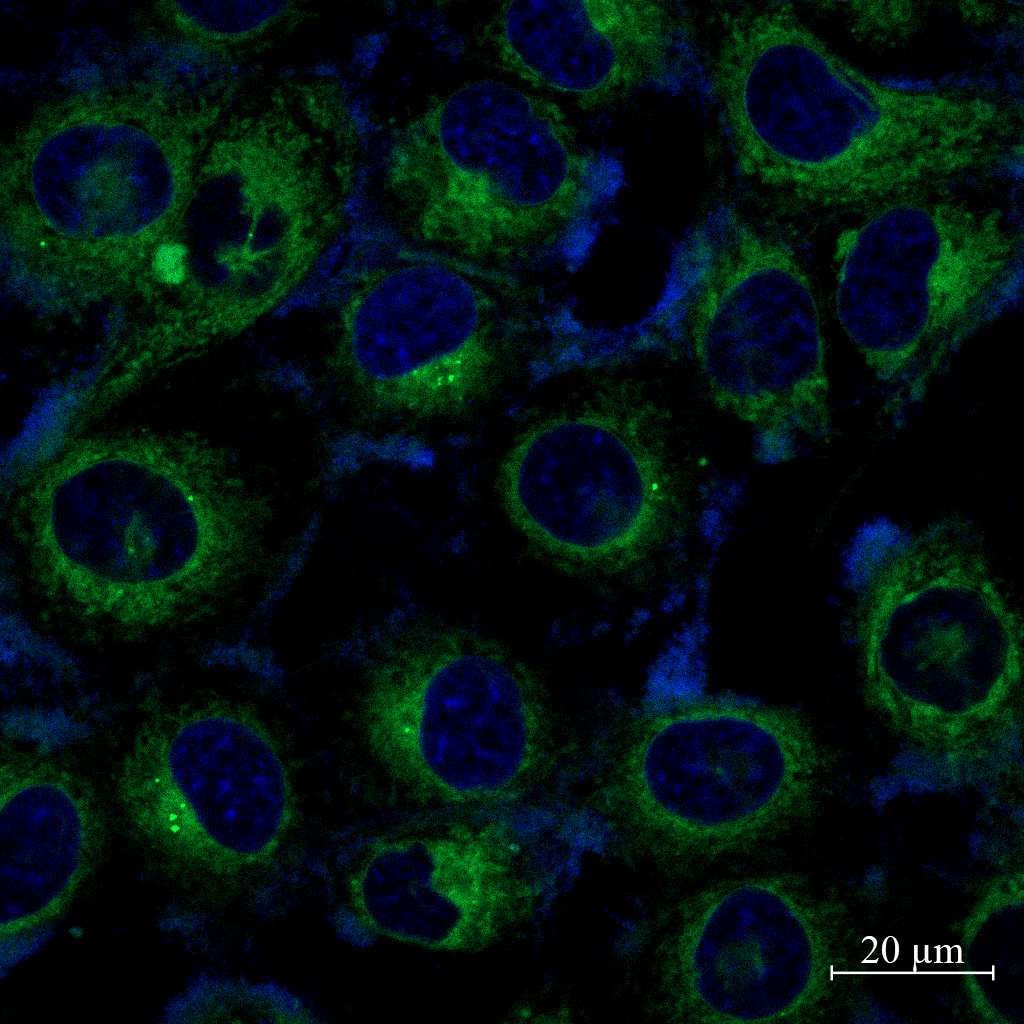

Supplement: Supplementary file 1 [file vetsci-12-00147-s001.zip › raw data/fig5/C-Microscopy/repeat 1/WT-2/WT-2.tif]

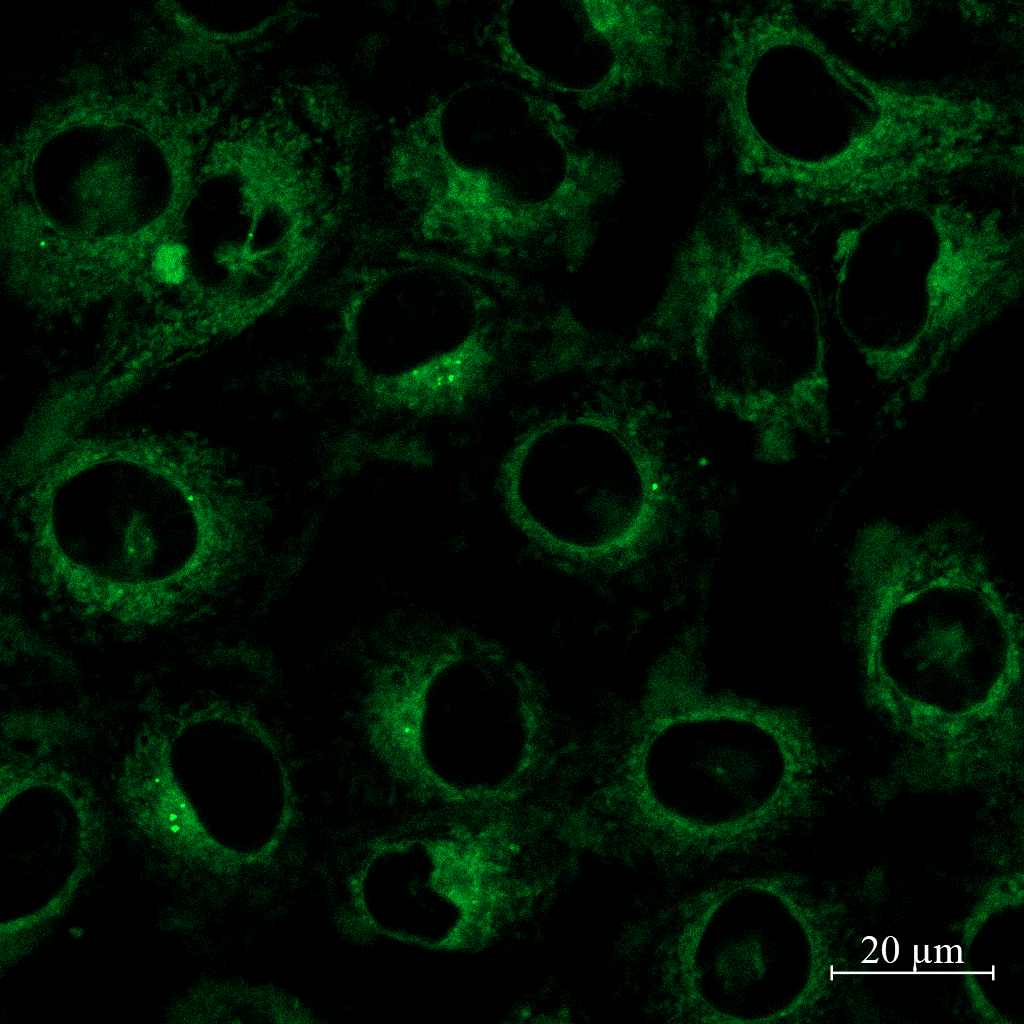

Supplement: Supplementary file 1 [file vetsci-12-00147-s001.zip › raw data/fig5/C-Microscopy/repeat 1/WT-2/WT-2_AF488-T1.tif]

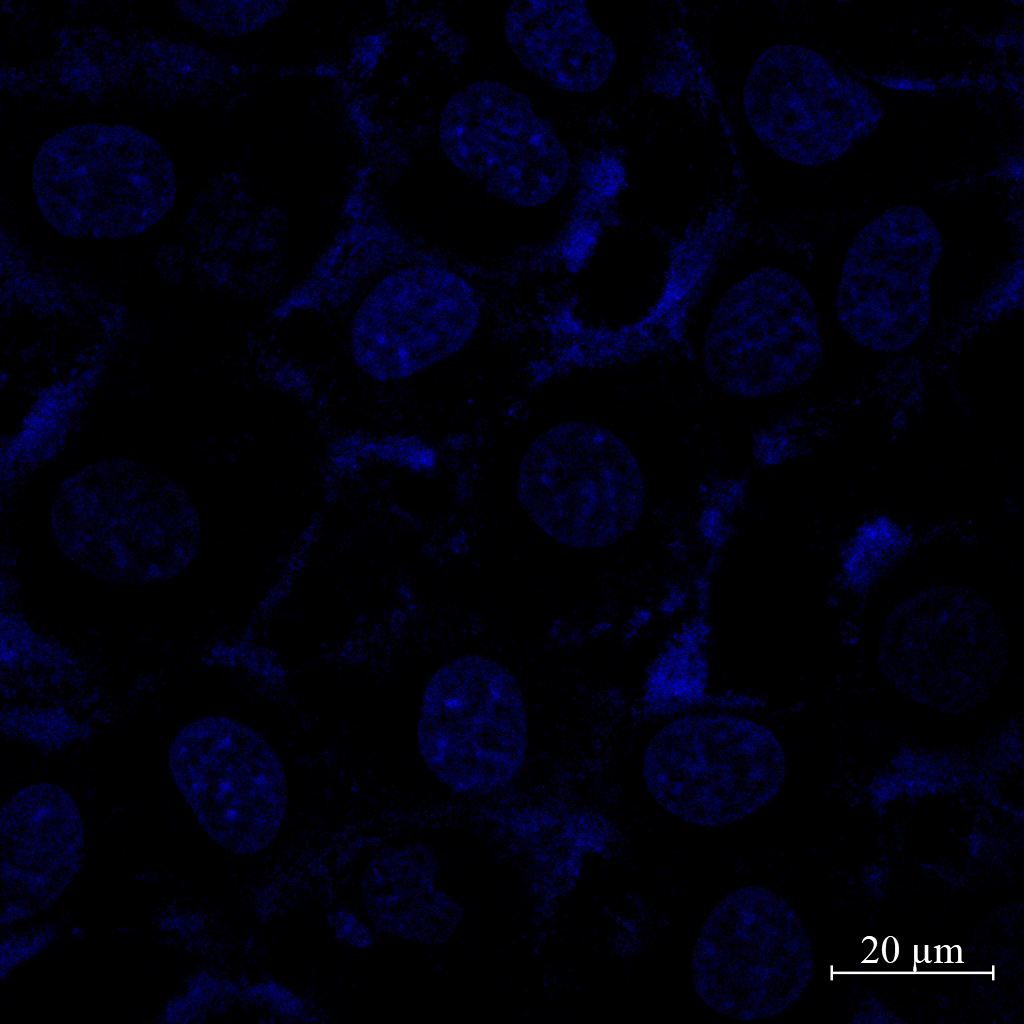

Supplement: Supplementary file 1 [file vetsci-12-00147-s001.zip › raw data/fig5/C-Microscopy/repeat 1/WT-2/WT-2_DAPI-T2.tif]

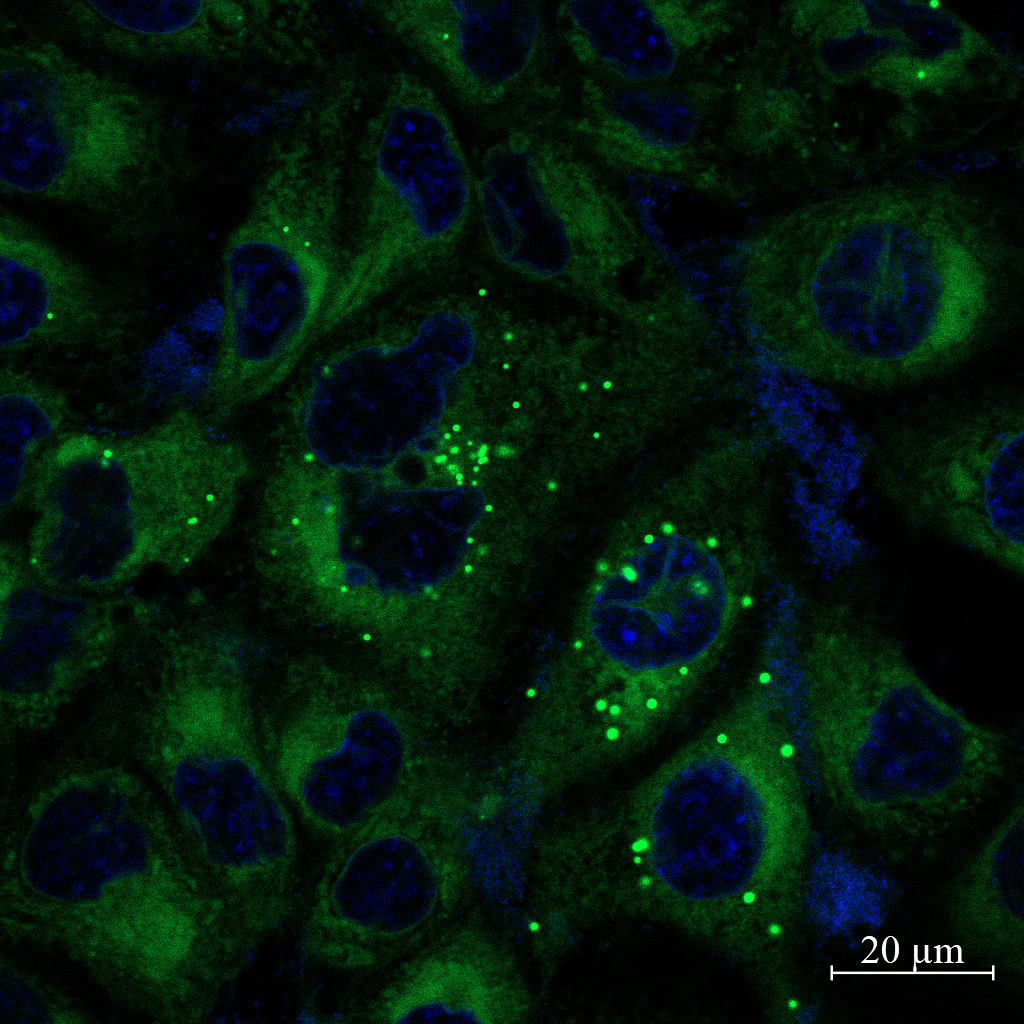

Supplement: Supplementary file 1 [file vetsci-12-00147-s001.zip › raw data/fig5/C-Microscopy/repeat 2/STEAP3-3/STEAP3-3.tif]

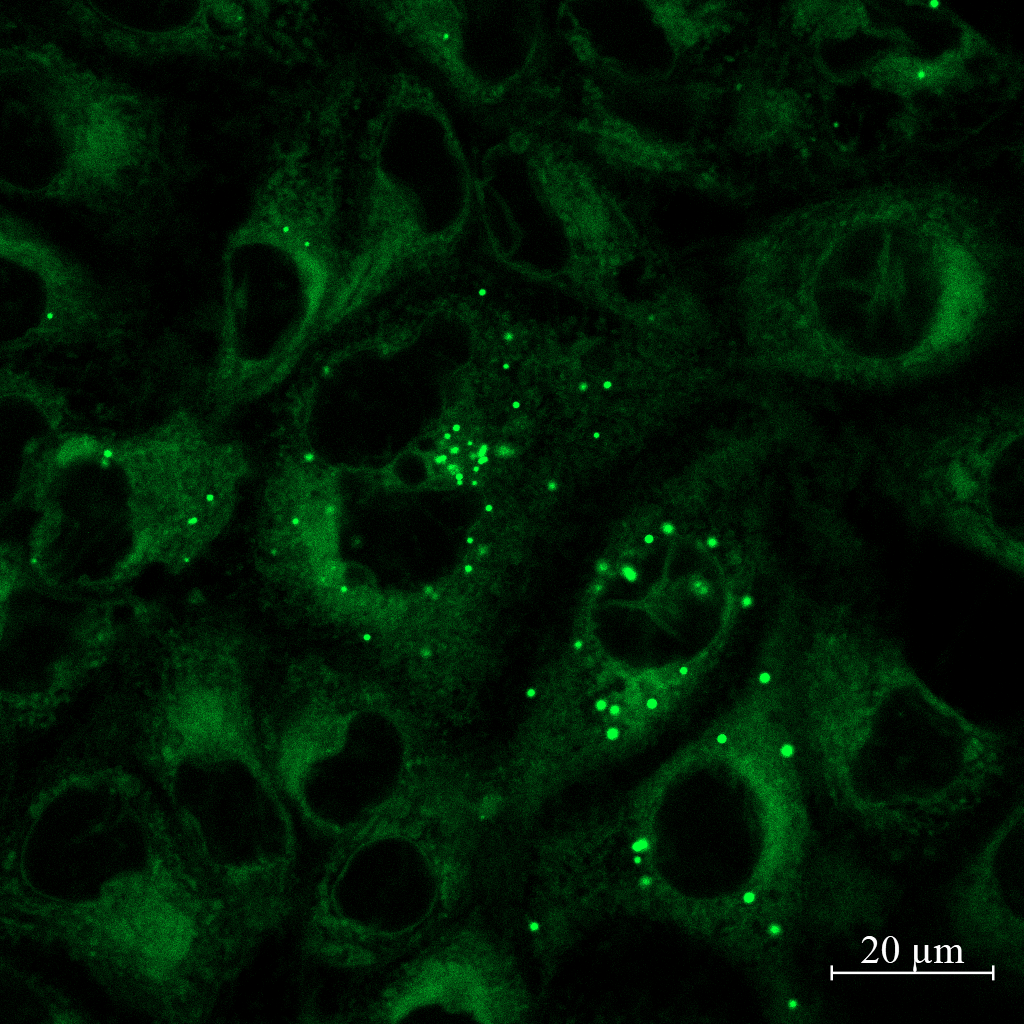

Supplement: Supplementary file 1 [file vetsci-12-00147-s001.zip › raw data/fig5/C-Microscopy/repeat 2/STEAP3-3/STEAP3-3_AF488-T1.tif]

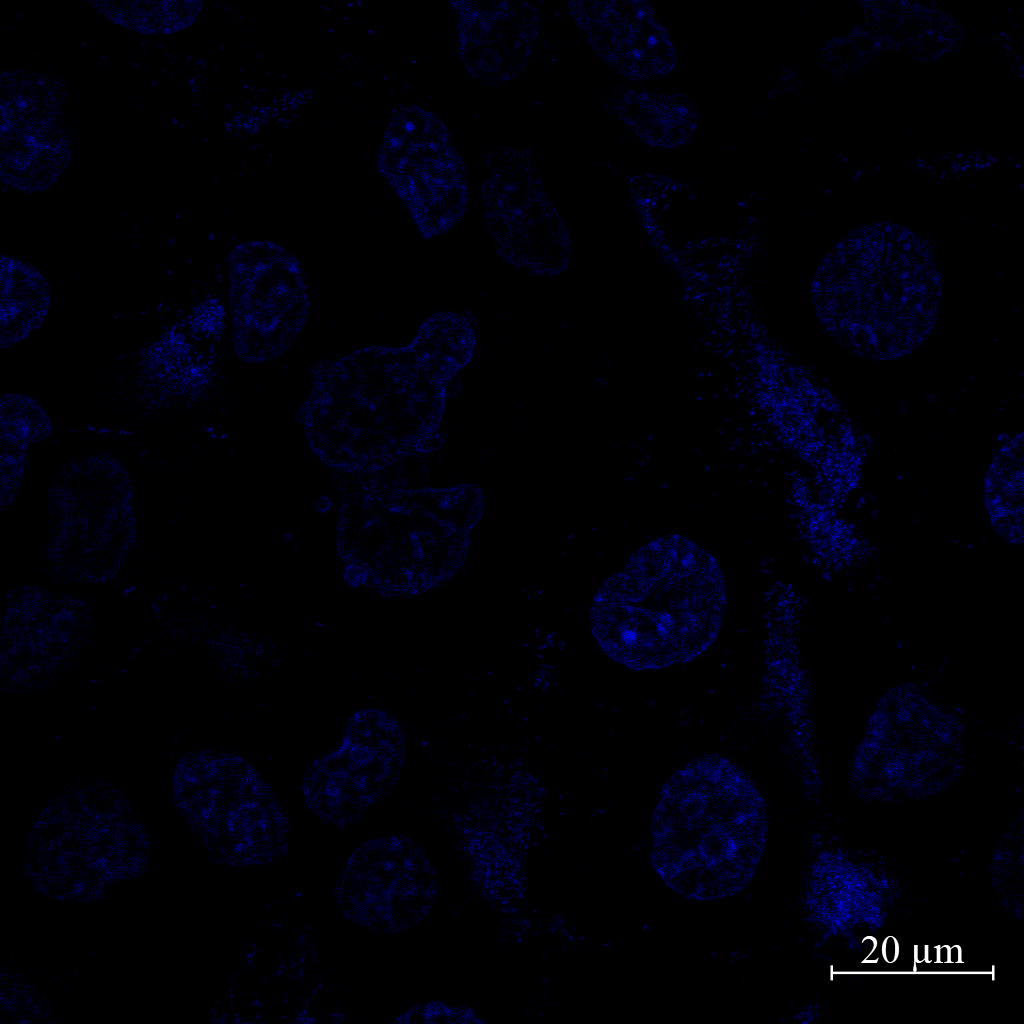

Supplement: Supplementary file 1 [file vetsci-12-00147-s001.zip › raw data/fig5/C-Microscopy/repeat 2/STEAP3-3/STEAP3-3_DAPI-T2.tif]

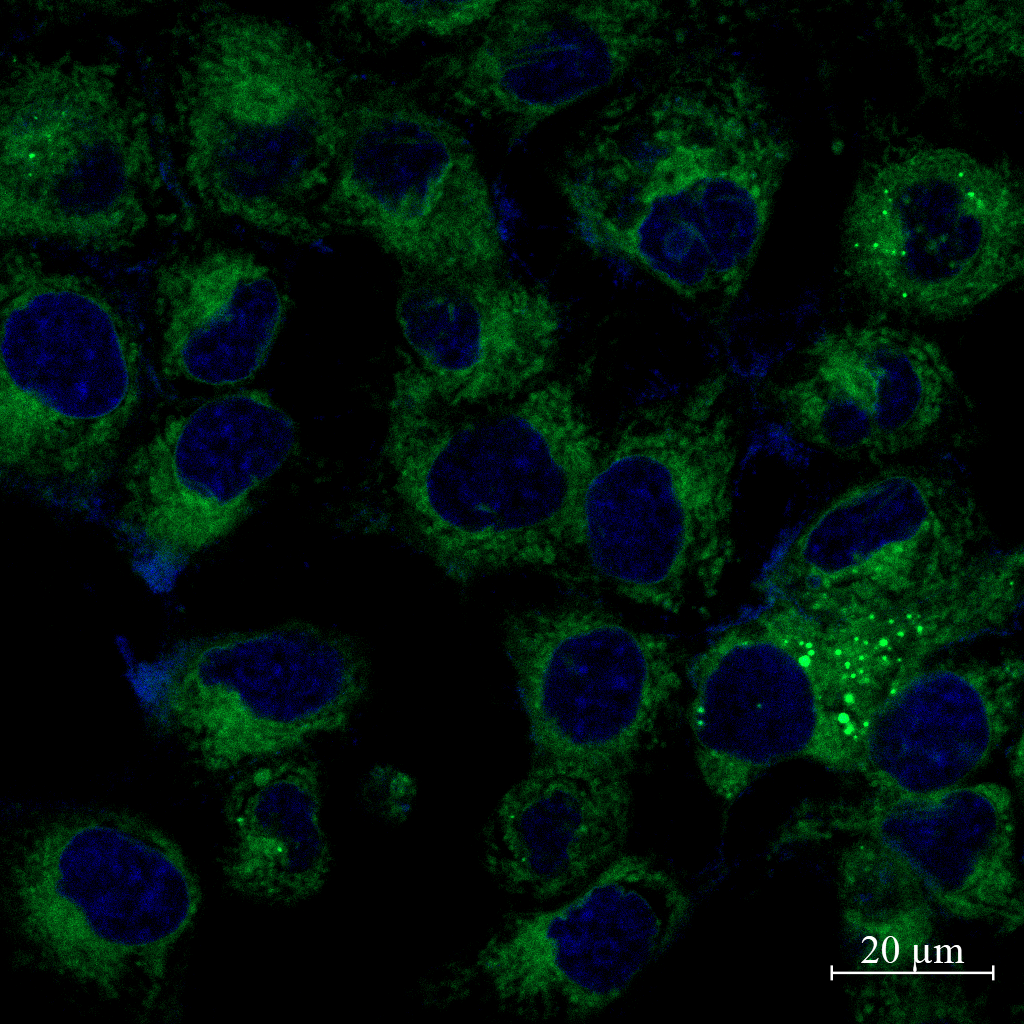

Supplement: Supplementary file 1 [file vetsci-12-00147-s001.zip › raw data/fig5/C-Microscopy/repeat 2/WT-3/WT-3.tif]

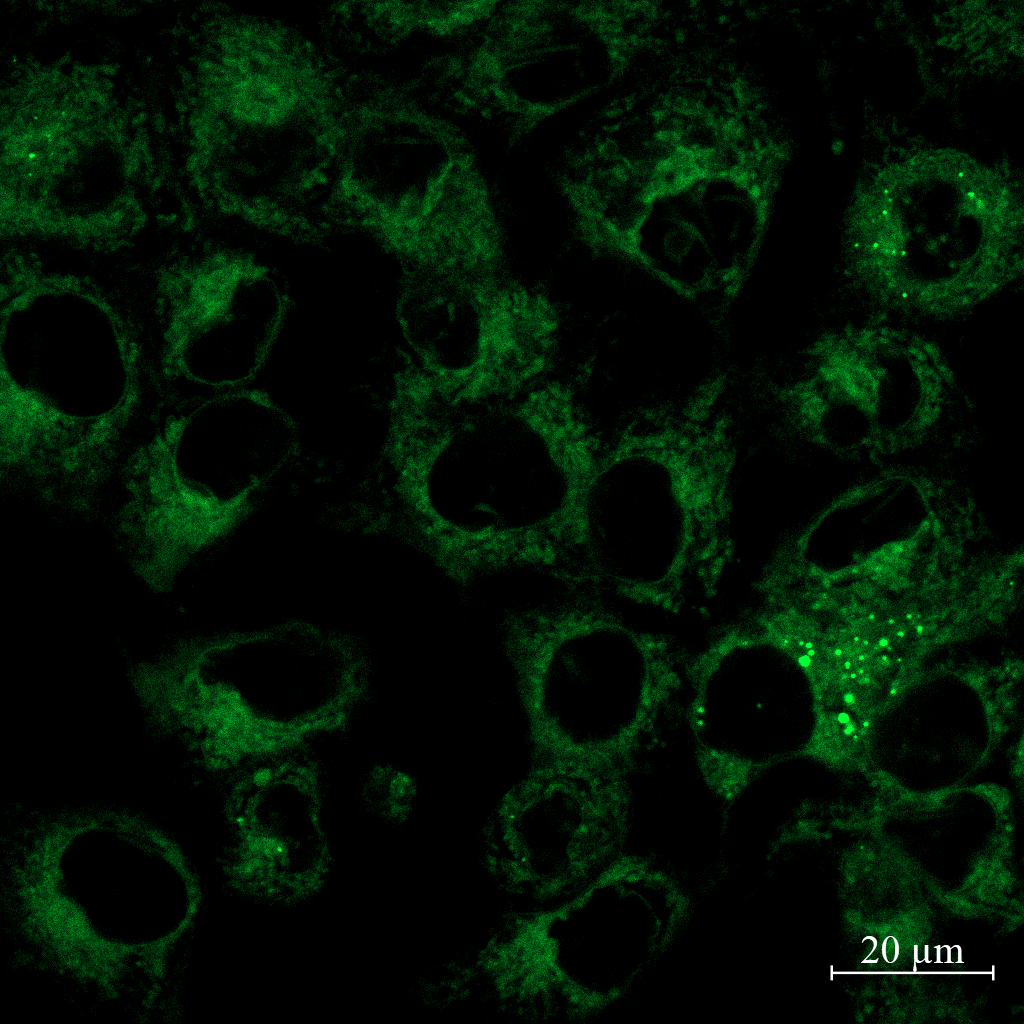

Supplement: Supplementary file 1 [file vetsci-12-00147-s001.zip › raw data/fig5/C-Microscopy/repeat 2/WT-3/WT-3_AF488-T1.tif]

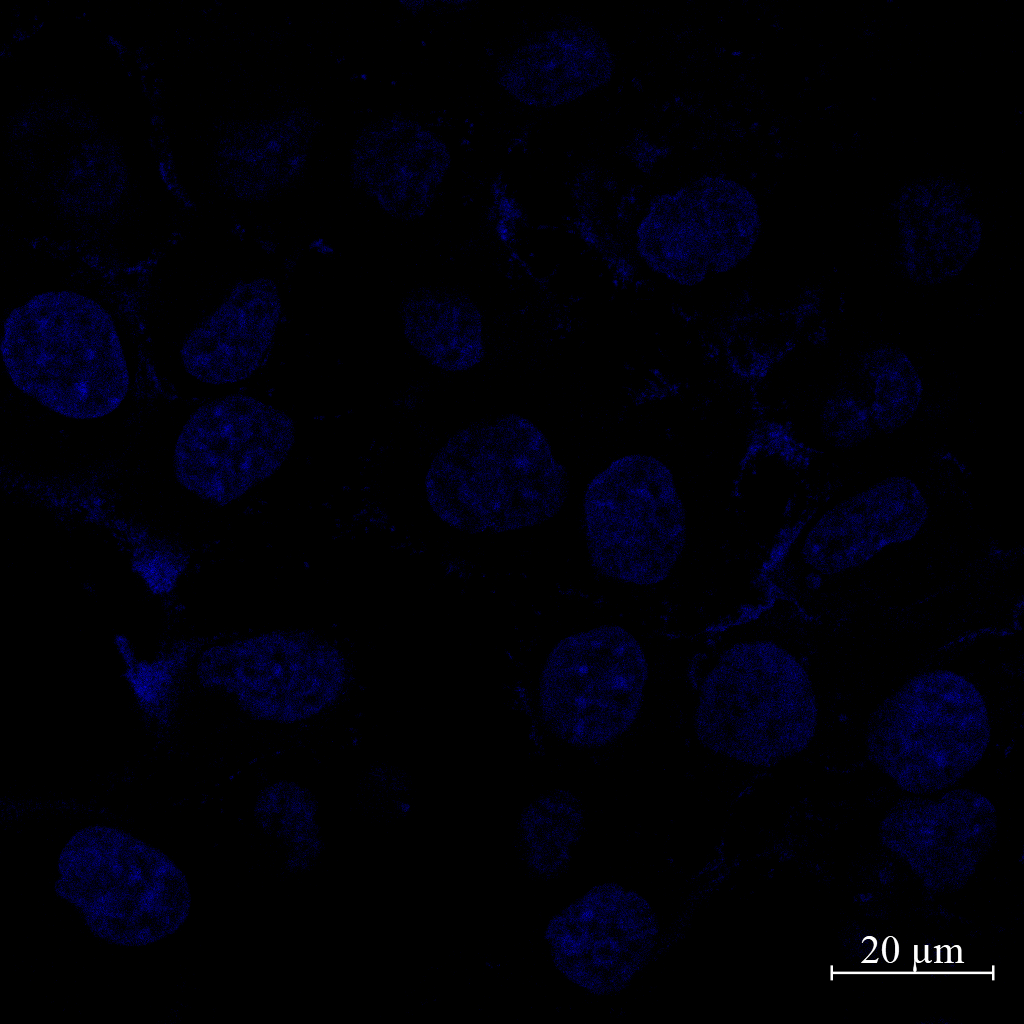

Supplement: Supplementary file 1 [file vetsci-12-00147-s001.zip › raw data/fig5/C-Microscopy/repeat 2/WT-3/WT-3_DAPI-T2.tif]
